# Unveiling the Electrocatalytic Activity of the GdBa_0.5_Sr_0.5_Co_2–*x*_Cu_*x*_O_5+δ_ (*x* ≥ 1) Oxygen Electrodes for Solid Oxide Cells

**DOI:** 10.1021/acsami.3c08667

**Published:** 2023-08-09

**Authors:** Keyun Li, Konrad Świerczek, Piotr Winiarz, Agnieszka Brzoza-Kos, Anna Stępień, Zhihong Du, Yang Zhang, Kun Zheng, Kacper Cichy, Anna Niemczyk, Yevgeniy Naumovich

**Affiliations:** † Faculty of Energy and Fuels, AGH University of Science and Technology, al. A. Mickiewicza 30, 30-059 Krakow, Poland; ‡ AGH Centre of Energy, AGH University of Science and Technology, ul. Czarnowiejska 36, 30-054 Krakow, Poland; § School of Materials Science and Engineering, University of Science and Technology Beijing, Beijing 100083, China; ∥ Key Laboratory of Advanced Fuel Cells and Electrolyzers Technology of Zhejiang Province, Ningbo Institute of Material Technology and Engineering, Chinese Academy of Sciences, Ningbo 315201, China; ⊥ Center for Hydrogen Technologies (CTH2), Institute of Power Engineering, ul. Augustowka 36, 02-981 Warsaw, Poland; # Institute of Power Engineering, ul. Mory 8, 01-330 Warsaw, Poland

**Keywords:** Cu-containing double perovskites, physicochemical properties, electrode polarization resistance, distribution of relaxation times analysis, reversible solid oxide cells

## Abstract

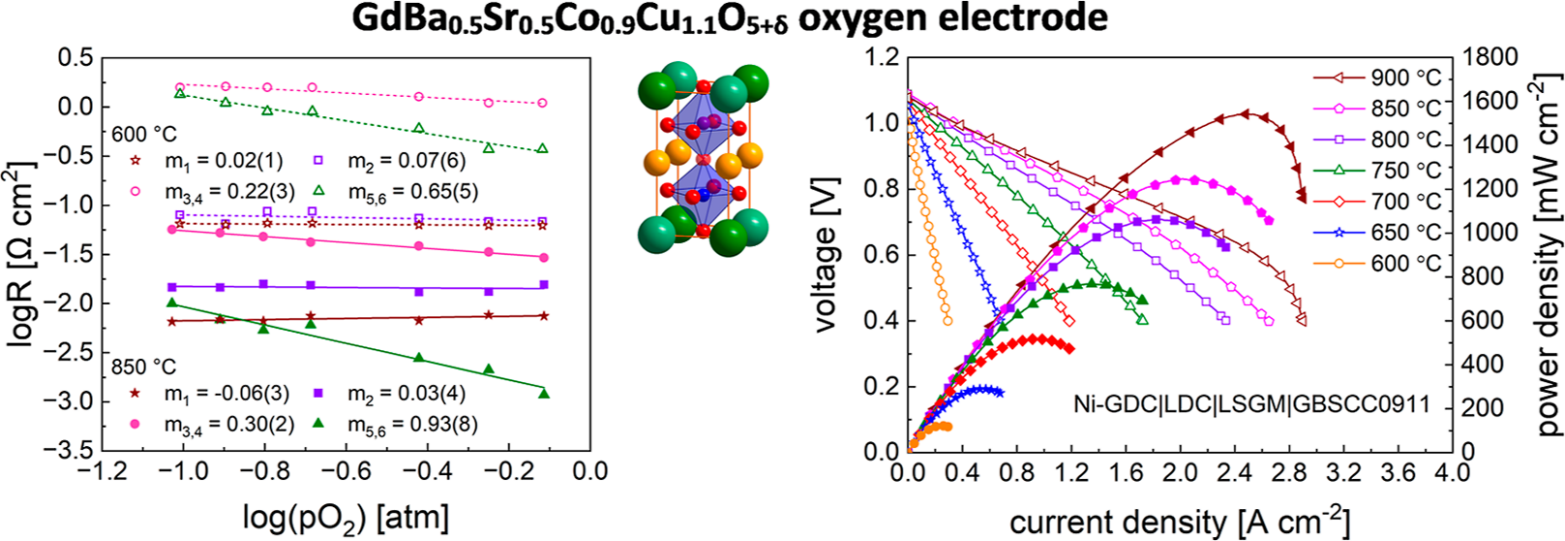

The A-site cation-ordered GdBa_0.5_Sr_0.5_Co_2–*x*_Cu_*x*_O_5+δ_ (GBSCC) double perovskites are evaluated regarding the development of high-performance oxygen electrodes for reversible solid oxide cells (rSOCs). The aims are to maximally decrease the content of toxic and expensive cobalt by substitution with copper while at the same time improving or maintaining the required thermomechanical and electrocatalytic properties. Studies reveal that compositions with 1 ≤ *x* ≤ 1.15 are particularly interesting. Their thermal and chemical expansions are decreased, and sufficient transport properties are observed. Complementary density functional theory calculations give deeper insight into oxygen defect formation in the considered materials. Chemical compatibility with La_0.8_Sr_0.2_Ga_0.8_Mg_0.2_O_3−δ_ (LSGM) and Ce_0.9_Gd_0.1_O_2−δ_ (GDC) solid electrolytes is evaluated. It is documented that the GdBa_0.5_Sr_0.5_Co_0.9_Cu_1.1_O_5+δ_ oxygen electrode enables obtaining very low electrode polarization resistance (*R*_p_) values of 0.017 Ω cm^2^ at 850 °C as well as 0.111 Ω cm^2^ at 700 °C, which is lower in comparison to that of GdBa_0.5_Sr_0.5_CoCuO_5+δ_ (respectively, 0.026 and 0.204 Ω cm^2^). Systematic distribution of relaxation times analyses allows studies of the electrocatalytic activity and distinguishing elementary steps of the electrochemical reaction at different temperatures. The rate-limiting process is found to be oxygen atom reduction, while the charge transfer at the electrode/electrolyte interface is significantly better with LSGM. The studies also allow elaborating on the catalytic role of the Ag current collector as compared with Pt. The electrodes manufactured using materials with *x* = 1 and 1.1 permit reaching high power outputs, exceeding 1240 mW cm^–2^ at 850 °C and 1060 mW cm^–2^ at 800 °C, for the LSGM-supported cells, which can also work in the electrolysis mode.

## Introduction

As a novel energy conversion technology, solid oxide cells (SOCs) have drawn considerable attention because of important advantages, including, among others, eco-friendly reversible operation, a wide range of fuel selection (for the fuel cell mode), and a high energy conversion efficiency.^1^ SOC-based generators can be applied not only in households but also in larger-scale applications, such as commercial combined heat and power units.^2^ A reversible mode of rSOCs enhances operational flexibility, as in the electrolysis mode, hydrogen can be produced (and later stored) utilizing surplus electricity. This is very beneficial for the functioning of the power grid.^3^ The features mentioned above may turn rSOC-type generators into multifunctional energy conversion devices in the near future.

Among the crucial components in each SOC, a cathode plays a critical role in terms of operation and lifespan. La_1–*x*_Sr_*x*_MnO_3_ (LSM), which is a reliable and conventional cathode material, has been adopted for a long time already. Nevertheless, it suffers from poor oxygen ion mobility and insufficient catalytic activity at intermediate and lower temperatures (<800 °C). The electrochemical reaction at the LSM oxygen electrode is limited only to the triple phase boundary (TPB) region between the electrode, electrolyte, and gas phase. To overcome this drawback, various mixed ionic-electronic conductors (MIEC) have been introduced. The past few years have shown intensive research focused on finding new suitable materials. With plenty of studies carried out, different perovskite-type and related compounds have been proven as promising and effective candidates, like, e.g., La_1–*x*_Sr_*x*_Co_1–*y*_Fe_*y*_O_3−δ_ (LSCF),^4−6^ Ba_1–*x*_Sr_*x*_Co_1–*y*_Fe_*y*_O_3−δ_ (BSCF),^6,7^ Sm_1–*x*_Sr_*x*_CoO_3−δ_,^6,8^ and K_2_NiF_4±δ_-type oxides (Ruddlesden–Popper structure).^4,9^ The progress resulted in a practical reduction of the operating temperature of rSOCs, which is of critical importance for lowering operating costs.

### Double Perovskite-Type Oxides

Among candidate oxygen electrode materials, 1:1 A-site layered AA′B_2_O_5+δ_ (A: rare-earth element, A′: alkaline earth metal, typically Ba, B: 3d metal) perovskites have drawn much attention because of their unique layered structure. In such materials, it is common to write the total oxygen content as 5 + δ (0 ≤ δ ≤ 1), which indicates that the total molar amount of the oxygen vacancies in the structure is 1 – δ. Due to a large radii difference between the A-site and A′-site elements, an ordered structure with AO_δ_ and A′O layers alternating along the *c*-axis is typically formed.^10^ The oxygen vacancies are preferentially located at AO_δ_ layers, forming fast oxygen anion migration channels.^9,11,12^ Thus, such materials usually exhibit excellent, but anisotropic oxygen anion conductivity at elevated temperatures.^13^ Of importance, δ can be interpreted as indicating the oxygen content in the layer related to the smaller A-site cations crystallographic plane. However, the degree of the A–A′ order can sometimes not be very high, making the structural description even more complicated.^14^ As already marked, depending on the chemical composition, high oxygen deficiency may be observed in some compounds, with δ close to zero, for example, for YBaMn_2_O_5_.^15^ For Co-based double perovskites, the oxygen nonstoichiometry is also strictly related to the presence of mixed Co^3+/4+^ states, enabling excellent electronic transport. For such oxides, the valence electrons are largely delocalized, resulting in a very high total electrical conductivity. For example, for LnBaCo_2_O_5+δ_ (Ln: Pr, Nd, Sm, and Gd), σ values reach above 100 S cm^–1^ in the temperature range of 50–700 °C.^16^ Additionally, it can also be found that, e.g., for PrBaCo_2_O_5+δ_, the maximum total conductivity can exceed 1000 S cm^–1^.^17^ This is related to the actual oxygen vacancy concentration, as their presence influences both the ratio of Co^3+^/Co^4+^ and the effectiveness of the charge transport. Interestingly, considering the electronic component of total conductivity in a more general way, a small polaron conduction mechanism should be paid attention to, with a transfer of the electrons realized in “–B–O–B–” chains. This is especially evident when the B-site element can vary in its oxidation states and when the valence electrons are more localized (e.g., as for Mn).^17,18^ In this case, the presence of the oxygen vacancy interrupts charge transfer between two different B-site cations.

### Doping of LnBaCo_2_O_5+δ_ Double Perovskites

Overall, the Co-containing double perovskite-type materials can be regarded as very promising cathode materials for rSOCs based on their exceptional mixed ionic-electronic conductivity and high oxygen reduction reaction (ORR) catalytic activity.^12,19,20^ Nevertheless, the discussed compounds exhibit several drawbacks, which are basically associated with high values of the thermal expansion coefficient. For example, in LnBaCo_2_O_5+δ_ (Ln: La,^21^ Pr,^22^ Nd,^23^ Sm,^24^ Gd^23^) series, TEC can exceed 20 × 10^–6^ K^–1^, and low redox stability can also be observed, which is mainly related to the high Co content. However, with similar flexibility regarding possible doping as in the case of simple perovskites, introducing other, especially 3d metal elements, can be used to mitigate these drawbacks. For example, plenty of works that yielded positive effects are reported, showing substitution with Fe,^18,22,23^ Ni,^25^ Mn,^26^ and Cu^18,27−30^ at the cobalt site. While not the most common, copper doping can be regarded as an effective and interesting option. It has a positive effect on decreasing TEC values, but it also results in lowered total conductivity.^18,29,31,32^ For example, Zhang et al.^31^ reported that Cu-doping at the B-site of YBaCo_2_O_5+δ_ causes a decrease in TEC from 17.8 × 10^–6^ K^–1^ for YBaCo_2_O_5+δ_ to 14.7 × 10^–6^ K^–1^ for YBaCo_1.4_Cu_0.6_O_5+δ_ in the range of 30–850 °C. The reported electrode polarization resistance (*R*_p_) was also good, 0.041 Ω cm^2^ at 800 °C for YBaCo_1.4_Cu_0.6_O_5+δ_. While the *R*_p_ is somewhat higher than that for the Cu-free counterpart, it is still more than acceptable. A similar phenomenon could also be seen in the study carried out by Kim and Manthiram,^30^ which obtained a lowered TEC of NdBaCoCuO_5+δ_ and GdBaCoCuO_5+δ_ (16.4 × 10^–6^ and 14.5 × 10^–6^ K^–1^, respectively) as compared to their cobalt-pure counterparts (20.7 × 10^–6^ and 19.7 × 10^–6^ K^–1^) in the range of 80–900 °C. At the same time, as with most Cu-containing oxides, the melting point of the material is lowered, which may be beneficial for preparation of the electrode layers at lower temperatures but may also influence the possibility of synthesizing pure compounds. It should also be mentioned that replacing Co with other elements is usually associated with lower total conductivity.^33,34^

Meanwhile, the partial substitution of Ba with Sr at the A′-site in double-perovskite oxides has promising effects on modifying the properties of the cathode since Sr can reduce the crystal distortion due to its smaller ionic radius as compared to Ba^2+^. Also, through the increased oxygen content, it results in enhanced small polaron hopping routes (better −B–O–B– transport).^20,35,36^ Jun et al.^19^ reported their enhanced conductivity of high Sr-content (SmBa_0.25_Sr_0.75_Co_2_O_5+δ_, almost above 1000 S cm^–1^), apparently higher than its Sr-free counterpart in the range of 100–700 °C (SmBaCo_2_O_5+δ_, around 600 S cm^–1^). Moreover, West and Manthiram^28^ obtained an increased maximum electrical conductivity of NdBa_0.5_Sr_0.5_CoCuO_5+δ_ (over 350 S cm^–1^ at 300 °C) compared to that of NdBaCoCuO_5+δ_ (around 100 S cm^–1^ at 900 °C). Generally, it can also be expected that improved conductivity results also in enhanced electrochemical properties. Yoo et al.^36^ conducted a study of NdBa_0.25_Sr_0.75_Co_2_O_5+δ_ and NdBa_0.5_Sr_0.5_Co_2_O_5+δ_ combined with GDC as composite cathodes, which could reach lower *R*_p_ values of 0.116 and 0.112 Ω cm^2^ at 600 °C, respectively, as compared to their Sr-free counterparts at the same temperature. Similarly, SmBa_0.25_Sr_0.75_Co_2_O_5+δ_ demonstrated a decreased *R*_p_ of 0.138 Ω cm^2^, compared to 0.192 Ω cm^2^ of SmBaCo_2_O_5+δ_ at 600 °C.^19^ The same enhancement was also reported in different studies, like for NdBa_0.25_Sr_0.75_CoCuO_5+δ_^28^ and PrBa_0.25_Sr_0.75_CoCuO_5+δ_.^20^ Nevertheless, knowledge about the similar doping effect of Sr has not been sufficiently investigated yet for high Cu-content double perovskites. However, a high content of Sr may result in an increased TEC, which is an undesirable effect.

Regarding the choice of the A-site rare-earth elements, larger cations cause more oxygen to be present in the materials but also higher thermal expansion, as is observed for Co-containing double perovskites.^21,23,28^ At the same time, small elements may decrease the chemical stability of the compounds. It can therefore be stated that the choice of the intermediate-size rare-earth element appears beneficial, and this can also be applied to the Cu-doped double perovskites.

Considering the discussion given above, doping with a relatively high amount of Cu (*x* ≥ 1), and selecting Gd, as well as 1:1 substitution of Ba by Sr, all seem like a reasonable approach for developing high-performance double-perovskite oxygen electrode materials for rSOCs. Consequently, in this work, we focus on the interesting, but less studied substitution with Cu in the GdBa_0.5_Sr_0.5_Co_2–*x*_Cu_*x*_O_5+δ_ series. Initially, the research was undertaken to establish the solid solution formation range as well as optimize the content of the economically-favorable and environmentally-benign copper (minimize Co content). While the introduction of a higher content of copper is expected to lower the TEC values of the compounds, it is unknown how different Co/Cu ratio values would influence the electrocatalytic activity. A strong emphasis was put on the systematic evaluation of the polarization resistance and the charge transfer in the case of GBSCC electrodes manufactured on LSGM and GDC solid electrolytes, as well as with different current collectors (Pt or Ag). Furthermore, in the case of the best-performing GdBa_0.5_Sr_0.5_Co_0.9_Cu_1.1_O_5+δ_ oxygen electrode, the conducted DRT analyses allowed unveiling of the rate-limiting step of the electrochemical reaction.

## Experimental Section

### Sample Preparation

The considered series of GdBa_0.5_Sr_0.5_Co_2–*x*_Cu_*x*_O_5+δ_ (0 ≤ *x* ≤ 2) oxides were obtained by a sol–gel method combined with the self-combustion step. The naming scheme of the samples was adopted that, e.g., GBSCC0911 notation corresponds to GdBa_0.5_Sr_0.5_Co_0.9_Cu_1.1_O_5+δ_ composition. Initially in the process, CuO (99+% purity) was dissolved in a diluted solution of nitric acid. After that, the respective amounts of barium, strontium, and cobalt nitrates (all above 99.9% purity) were added, with the solution mixed on the heating magnetic stirrer. Then, citric acid as well as ethylenediaminetetraacetic acid were added in a 1.5:1 ratio to all metal cations and in a 1:1 ratio with respect to the total amount of Ba^2+^ and Sr^2+^ cations, respectively. The pH value was adjusted to around 7–8 using an ammonia solution. During heating, subsequent water evaporation, sol formation, and transition into a gel could be observed. With further increase in temperature, autoignition occurred at ca. 200–300 °C, thanks to the presence of ammonium nitrate (reaction ignitor). This, as well as the oxidation of carbon, generated a dark ash, which was then ground in an agate mortar. The initial calcination was conducted at 400 °C for 2 h in air. The second step was done at 800 °C for 6 h, also in the air. In the pellet preparation process, respective GBSCC materials were pressed at ca. 100 MPa in a 13 mm matrix and then heat-treated at 1000 °C for 6 h.

### Density Functional Theory Calculations

The quantum mechanical calculations based on density functional theory (DFT) were performed using the VASP 6 package (Vienna Ab Initio Simulation Package). General Gradient Approximation + U with the Perdew–Burke–Ernzerhof exchange–correlation functional was applied (GGA-PBE). Accurate precision with an increased plane-wave cutoff energy of 520 eV for cell optimizations was maintained. The convergence energy was set to 1.0 × 10^–5^ eV per atom and 0.02 eV Å^–1^ for force, using the blocked Davidson algorithm. The selected U values for Co and Cu were taken from the literature as 3.0 and 0.0 eV, respectively.^37,38^ The structural optimizations were performed on 2 × 2 × 1 supercells of the GdBa_1–*x*_Sr_*x*_Co_2–2*y*_Cu_2*y*_O_5+δ_ double-perovskite systems, where *x* = 0, 0.5, and 1; *y* = 0, 0.5, and 1; δ = 0, 0.25, 0.5, 0.75, and 1. The exemplary supercell is shown in Figure S1 (middle part). Bearing in mind that there are many structural possibilities to replace barium with strontium and cobalt with copper, as well as that the respective supercell models of the doped materials should not be too big or simple, energetically favorable (i.e., yielding more negative total DFT energy) configurations were chosen. In the case of barium, which is present only in the (002) plane, it was replaced by strontium diagonally. Cobalt atoms occupy two planes; hence, there are more ways to replace them with copper. These atoms were replaced alternately in each perpendicular direction, as schematically shown in Figure S1 (left and right parts). For calculations of the energy of oxygen vacancy formation, *E*_ox.vac._, the following basic formula was used
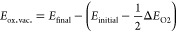
1where *E*_final_ is the energy of the final system, *E*_initial_ is the energy of the initial system, and Δ*E*_O2_ is the total energy of a free oxygen molecule, taken from the literature as Δ*E*_O2_ = −9.95 eV.^39^ In most of the calculations regarding the oxygen content, it was taken in the initial material as 6 (δ = 0), and the final content was 5.75, 5.5, 5.25, or 5. Consequently, the value of Δ*E*_O2_ was appropriately multiplied to reflect the actual oxygen content difference.

### Characterization of the Physicochemical Properties and Chemical Stability of GBSCC

X-ray diffraction (XRD) measurements were carried out to study the crystal structure of the considered GdBa_0.5_Sr_0.5_Co_2–*x*_Cu_*x*_O_5+δ_. The experiments were performed at RT in a range of 10–100° on a Panalytical Empyrean diffractometer. Cu Kα radiation was used, and the detector was a PIXcel3D. High-temperature XRD (HT-XRD) studies were also performed in the air from RT to 1000 °C, on heating and cooling. For this, the Anton Paar HTK 1200N oven chamber was adopted. For analysis of the structural data at RT, GSAS II software^40^ was used, with Rietveld refinements of the diffractograms. The same approach was utilized for results from high temperatures, based on which linearized TEC was derived. For comparison, the thermal expansion of the dense GdBa_0.5_Sr_0.5_Co_2–*x*_Cu_*x*_O_5+δ_ ceramics was studied up to 900 °C in the air using a Linseis L75 Platinum Series dilatometer.

The morphology and chemical composition of the considered materials were investigated using scanning electron microscopy (SEM) with energy-dispersive X-ray spectroscopy (EDS) analysis using ThermoFisher Scientific Phenom XL Desktop SEM. The applied accelerating voltage was 15 kV.

To have a reference point regarding the total oxygen content in the studied materials at RT, the iodometric titration method was adopted.^41^ Initially, GBSCC powdered samples were equilibrated by slow cooling to RT after annealing at 800 °C beforehand. Then, the respective sample was dissolved in KI solution. Sodium thiosulfate solution was used as the titration agent on the EM40-BNC Mettler Toledo titrator equipped with a Pt electrode. The selected method was that the end point was determined by the color change of the solution (not the potential change). This approach was tested and found to be more reliable. At least three titration experiments were performed for each sample.

The respectively evaluated oxygen content in the GBSCC perovskites was used to obtain information about changes occurring with temperature. For this, thermogravimetric (TG) studies were performed on a TA Q5000 IR thermobalance. In a typical measurement, ca. 60 mg of the powder was placed in a Pt holder. Then, two successive heating/cooling cycles with a 2 °C min^–1^ rate were registered in the following atmospheres: initially in air, then in oxygen, again in the air, in 1 vol. % O_2_ in Ar, pure Ar, as well as in 5 vol. % H_2_ in Ar. The final stage involved the decomposition of the materials. It should be noted that the titration result was adopted as the starting point of the second heating run of the second (i.e., after O_2_) air measurements.

The total electrical conductivity of GdBa_0.5_Sr_0.5_Co_2–*x*_Cu_*x*_O_5+δ_ was evaluated in the air by the pseudo-4-probe DC method. The used sinters were dense and of a rectangular shape (approx. 7 × 4 × 1.5 mm). Platinum current collectors were prepared by applying Pt paste to the opposite faces of the specimens. The sintering of Pt was done at 850 °C for 15 min. In the custom setup, the ProboStat holder (NorECs) was used to mount the samples, while data were acquired with a Keithley 2000 (Tektronix) multimeter. Also, Omega2 (NorECs) software was used for data recording.

GdBa_0.5_Sr_0.5_Co_2–*x*_Cu_*x*_O_5+δ_ materials were also evaluated in terms of their chemical compatibility with La_0.8_Sr_0.2_Ga_0.8_Mg_0.2_O_3−δ_ (LSGM, FuelCellMaterials) and Ce_0.9_Gd_0.1_O_2−δ_ (GDC, Cerpotech) solid electrolytes. In the experiments, the respective cathode and electrolyte powders were mixed in a 1:1 wt ratio and then annealed in the air for 2 h at 950 °C (with LSGM) and 900 °C (with GDC). The electrolyte materials were preliminarily heat-treated at 1450 °C (LSGM) and 1350 °C (GDC). Then, XRD studies were conducted at RT on the heat-treated mixtures.

### Fabrication and Tests of GBSCC-Based Symmetrical Cells and LSGM-Supported SOCs

Different symmetrical GBSCC/LSGM/GBSCC or GBSCC/GDC/GBSCC cells were manufactured using dense LSGM or GDC pellets as the support. In the beginning, the LSGM powder was mixed and ground with 1 wt % of poly(-vinyl butyral-co-vinyl alcohol-co-vinyl acetate) additive. In the next step, the mixture was pressed into disks (ca. 100 MPa, 13 mm in diameter) and sintered in air at 1450 °C for 8 h. For GDC, the whole process was the same, but the sintering was done at 1350 °C. Before screen-printing the electrode layers, the thickness of the pellets was reduced to ca. 300 μm on sandpaper. The cathode paste was obtained using the respective GBSCC powder, organic binder (terpineol-based 311237 ink, FuelCellMaterials), and pore former (starch). The weight ratio was 2:1:0.1. The paste was then screen-printed on the LSGM or GDC disks with two layers on each side. The layers of the symmetrical cells were sintered at 900 °C.

The as-prepared symmetrical cells were applied not only to conventional electrochemical impedance but also to long-term tests. Because of that, different current collectors were used: Ag and Pt. For the silver, the Ag paste (FuelCellMaterials) was painted on both electrodes and sintered at 700 °C for 0.5 h in air. Additionally, Pt paste (ESL ElectroScience) was also adopted. It was sintered at 850 °C for 15 min in the air. Only small amounts of the respective metal pastes were used, and the formed shape was a mesh pattern.

For the full LSGM-supported cell tests, the thickness of the electrolyte support was reduced to ca. 200 μm on the machine grinder (Struers Labopol-30 with MD-Piano 220 diamond polishing plate). In the next step, the Ce_0.6_La_0.4_O_2−δ_ (LDC) buffer layer was deposited by screen printing on one side of the LSGM pellet and sintered at 1400 °C for 2 h. For LDC preparation, it was synthesized similarly as in ref (42), but the final sintering was at 1000 °C for 4 h in air. Regarding the fuel electrode preparation, the initial NiO/GDC wt. ratio was 3:2. The prepared slurry was screen-printed and sintered at 1350 °C for 2 h in air. After this, the selected GBSCC1010 and GBSCC0911 cathode pastes were respectively applied and sintered in the same conditions as explained above for the symmetric cells. As the current collector, Ag paste was used. The working area of the electrodes was ca. 0.28 cm^2^.

The properties of the symmetrical and full cells were characterized by utilizing electrochemical impedance spectroscopy (EIS). The setup used contained a Solartron 1260 frequency response analyzer and a Solartron 1287 electrochemical interface. The frequency range of the gathered signal was 0.1 Hz to 1 MHz. The perturbation amplitude was 25 mV. The temperature range of the studies for symmetrical cells was 600–850 °C, and for full cells, it was 600–900 °C. The measured spectra were typically fitted with an *L*-*R*_ohm_-(RQ)_HF_-(RQ)_MF_-(RQ)_LF_ equivalent circuit; however, for more simple curves, circuits with two or single (RQ) elements were also considered. The *R*_ohm_ indicates the total ohmic resistance of the electrolyte and electrodes, while the (RQ) components (arcs) represent different electrochemical processes occurring at high (HF), middle (MF), or low (LF) frequencies.^43,44^ The total electrode polarization resistance was calculated as the sum of all the resistance elements originating from the refined arcs. ZView and ZPlot (Scribner Associates) software were selected to gather data for EIS studies. It can be estimated that the relative error of *R*_p_ evaluation should not exceed 5%. Also, in order to get a better understanding of ORR steps for the selected GBSCC0911 electrode, the distribution of relaxation times analysis was carried out according to the standard procedures.^45,46^

Synthetic air flow (20 mL min^–1^) was applied in the studies of symmetrical cells. For the full cells, humidified (ca. 3 vol. % H_2_O) hydrogen was directed to the Ni-GDC electrode, and synthetic air flow was supplied to the GBSCC oxygen electrodes. The respective flow rates were 50 and 20 mL min^–1^. The cell was monitored from the open circuit voltage in the potentiodynamic mode by CorreWare and CorrView (Scribner Associates) software. The observed voltage and current density values were used to derive the power density output. Finally, the electrolysis mode tests were also conducted in the 600–750 °C temperature range. Since the electrode of interest was the oxygen electrode, the tests were performed with the same humidified (ca. 3 vol. % H_2_O) hydrogen supplied to the fuel electrode and synthetic air provided to the GBSCC electrode. The same gas flow conditions were applied.

## Results and Discussion

### Physicochemical Properties of GdBa_0.5_Sr_0.5_Co_2–*x*_Cu_*x*_O_5+δ_

**Figure 1:**
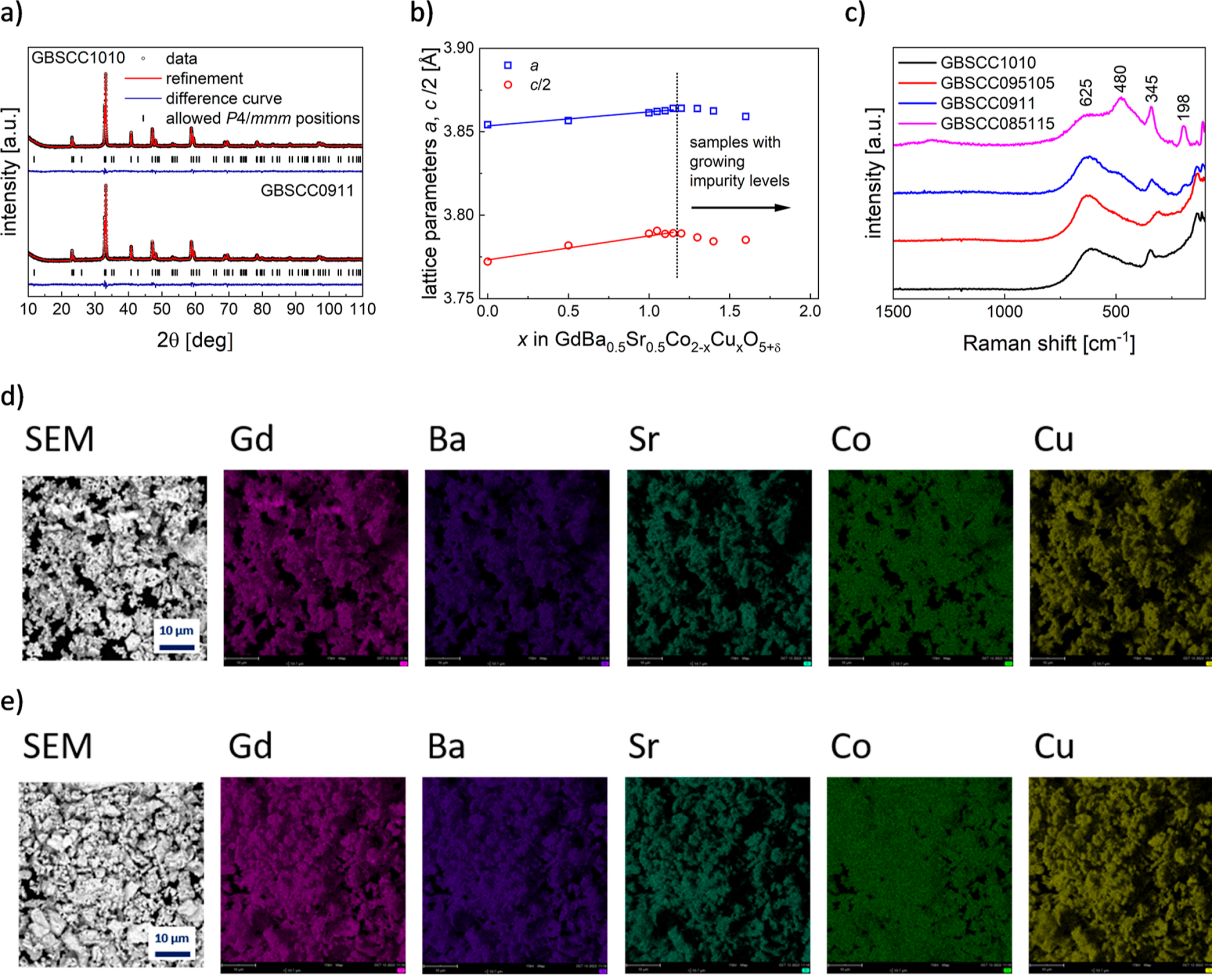
(a) XRD data for GBSCC1010 and GBSCC0911 samples recorded at RT together with Rietveld refinements. (b) Unit-cell parameters depend on the copper doping level in GdBa_0.5_Sr_0.5_Co_2–*x*_Cu_*x*_O_5+δ_ series. (c) Raman spectroscopy data for selected GBSCC materials. (d) Morphology of the GBSCC0911 and (e) GBSCC1010 powders, together with EDX elemental maps.

As can be seen in Figure 1a for the selected GdBa_0.5_Sr_0.5_CoCuO_5+δ_ (GBSCC1010) and GdBa_0.5_Sr_0.5_Co_0.9_Cu_1.1_O_5+δ_ (GBSCC0911) samples, XRD data recorded at RT indicate the presence of the desired A-site layered double-perovskite structure and tetragonal *P*4/*mmm* symmetry. The same crystal structure was observed for all other synthesized GBSCC materials, except samples with the assumed *x* exceeding 1.9. In this case, numerous additional peaks suggesting the presence of large amounts of different phases could also be seen on the registered diffractograms (Figure S2). Considering oxides have copper content in the range of 1.2 ≤ *x* ≤ 1.6, there is a secondary phase observed, which was identified as likely being orthorhombic triple perovskite. The material with *x* = 1.15 contains only a very minor amount of contamination.^47^ Despite re-sintering attempts in the same conditions, it was not possible to eliminate its presence (Table S1). Likely, a general influence of copper on the melting temperature of copper-containing oxides (i.e., a significant decrease in temperature) makes the synthesis more difficult.^36^ At the same time, decreasing oxygen content in the series with growing *x* also contributes to problems with obtaining phase-pure materials. It can be assumed that GdBa_0.5_Sr_0.5_Co_2–*x*_Cu_*x*_O_5+δ_ samples with high copper content are beyond the solid solution formation range.

Surprisingly, in the Cu content range of *x* = 0–1.15, the dependence of the *a* and (normalized) *c* unit cell parameters on the copper content, while close to linear, is rather insignificant (Figure 1b). Bigger changes were observed for similar double perovskites substituted with Fe,^22,23^ Mn,^26^ or Ni.^25^ The refined structural parameters are included in Table S1. The ionic radii of elements are as follows: 0.61 Å for Co^3+^ (high spin, 6-fold coordination) and 0.53 Å for Co^4+^ (high spin, 6-fold), 0.73 Å for Cu^2+^ (6-fold) and 0.54 Å for Cu^3+^ (low spin, 6-fold),^48^ as well as that δ is close to 0.5 (see below), it can be assumed that copper is introduced as a mixture of Cu^2+^ and Cu^3+^ (replacing Co^3+/4+^). This should also lead to a decrease in oxygen content. For samples with 1.2 ≤ *x* ≤ 1.2, the materials are somewhat contaminated, but the main phase shows unit cell parameters, which rather decrease with growing Cu content. This may be explained partially by the presence of the secondary phases but likely also indicates more complex structural changes.

Supplementary Raman spectroscopy studies revealed significant changes in the spectra with increasing copper content above *x* = 1 (Figure 1c). The most notable difference is related to the emergence of a signal at ca. 480 cm^–1^, as well as at ca. 198 cm^–1^. On the other hand, the main peak, centered at about 625 cm^–1^, is rather unaffected. According to the interpretation given in work^49^ for GdBaCo_2_O_5+δ_, the main signal (peak at 625 cm^–1^) can be interpreted as originating from bond stretching motions of oxygen connecting pyramids and octahedra in the *a*–*b* plane. At the same time, the 480 cm^–1^ peak appears to be related to the oxygen connecting only pyramids. Therefore, the ongoing changes indicate a significant rearrangement of the oxygen in the Gd-related layer. A model describing this effect is presented in Figure S3. This is likely the reason for a slightly non-monotonous behavior of the *c* parameter in the 1 ≤ *x* ≤ 1.15 range (Figure 1b), as well as possibly explaining the behavior of samples with copper content *x* > 1.15. The feature at ca. 345 cm^–1^ seems to be related to vibrations of Co/Cu; however, the origin of the emerging peak at ca. 198 cm^–1^ is currently unknown as it does not seem to be linked to the displacement of heavy ions, as discussed in.^49^

Importantly, as presented in Figure 1d,e, GBSCC1010 and GBSCC0911 samples show a high level of homogeneity at the microscale, with an even distribution of all cations visible and apparently a lack of segregation or precipitation of unwanted phases.

Considering the reasons discussed above for decreasing the Co content in the considered series of GdBa_0.5_Sr_0.5_Co_2–*x*_Cu_*x*_O_5+δ_, and taking into account the presence of secondary phases and hindered synthesis of materials with *x* > 1.15, GBSCC compositions with *x* = 1.0, 1.05, 1.1, and (negligibly contaminated) 1.15 were chosen for further studies.

**Figure 2:**
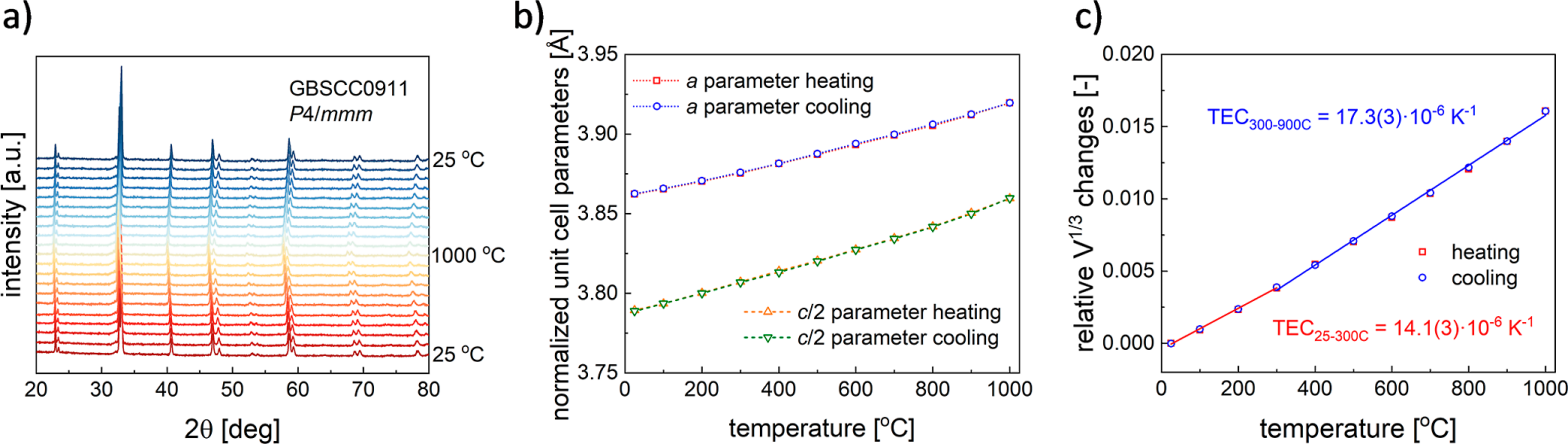
(a) HT-XRD data recorded for the GBSCC0911 material. (b) Derived unit-cell parameters’ dependence on temperature and (c) calculated TEC values.

With respect to the stability of as-synthesized compositions in a high-temperature range, consecutive XRD measurements were conducted up to 1000 °C and with cooling down to RT in the air (HT-XRD). As can be clearly seen in Figure 2a, GBSCC0911 maintains a stable tetragonal structure in the whole measured range without any phase transitions being detected. Moreover, other compositions also demonstrate the same good stability at high temperatures, as e.g., shown for GBSCC1010 in Figure S4a and for GBSCC095105 in Figure S4b. It can be concluded that the studied GBSCC compositions should be sufficiently stable not only during sintering of the oxygen electrode layers but also at the time of operation and testing of the cells.

Further information can be derived from the HT-XRD data as well. In Figure 2b (also Figure S4c,d), a monotonous and almost linear tendency can be observed for the temperature changes of not only the lattice parameter *a* but also for *c*. The increase in slope is not very significant but noticeable, marking the influence of the chemical expansion of the crystal lattice, adding to the physical one.^50,51^ The tendency of changes in the lattice parameters is maintained during heating and cooling, which further verifies the good stability in the high-temperature range of all the analyzed GBSCC samples. Since both unit cell parameters show similar behavior with temperature, TEC values were obtained from linearized changes in the unit cell volume. Selected data are presented in Figures 2c and S4e,f. A moderate thermal expansion behavior was detected for GBSCC0911, with TEC values of 17.3 × 10^–6^ and 14.1 × 10^–6^ K^–1^, respectively, in the ranges of 300–900 and 25–300 °C. Notably, there is almost no difference between data on heating and cooling. Compared to cobalt-based double-perovskite materials,^23,30^ but also with the GBSCC1010 sample from this study, it can be confirmed that Cu-doping allows successful optimization of the thermal expansion behavior (i.e., decrease of TEC values). The positive influence is visible in the whole temperature range, indicating limited chemical expansion. This is desirable for the manufacturing and operation of cells because of the mitigated thermomechanical mismatch.

Aiming to gain insight into the total oxygen content variation on Cu-doping in the considered GBSCC series, iodometric titration measurements were carried out to assess the average oxidation state of Cu and Co cations. Information about the derived oxygen content for the samples is included in Table S2, with a result for GBSCC1505 also added for comparison. It is evident that higher Cu-doping in samples with *x* ≥ 1 results in lower oxygen content at RT, but as already mentioned in the structural section, the actual values indicate the presence of mixed Co and Cu states for all materials. It can be summarized that for cobalt cations present in perovskite-type oxides, the average charge state is below +4, decreasing down to the more typical +3 state.^52^ For copper, it is usually below +3, with a +2 state often observed.^53^ Analyzing data from Table S2, it is evident that for GBSCC1010 the average charge state of Co and Cu is 3.14, likely indicating the presence of Co^3+/4+^ and Cu^2+/3+^ states. In the case of GBSCC0911, the average oxidation state of cobalt and copper is lower, 3.02, and decreases slightly below 3 for GBSCC085115. It still suggests the coexistence of different charge states of both cations but with a different ratio to maintain electroneutrality. The most probable explanation, which is in agreement with all the data, is that the oxidation state of Co is above +3.5, and for Cu, it is below +2.5, while both values somewhat decrease for higher Cu content. Also, it is not clear why the oxygen content for GBSCC1505 is lower than expected, taking into account the reported δ values for GdBa_0.6_Sr_0.4_Co_2_O_5.79_ and GdBa_0.4_Sr_0.6_Co_2_O_5.83_.^35^ More studies in this range of doping are needed to understand this effect.

**Figure 3:**
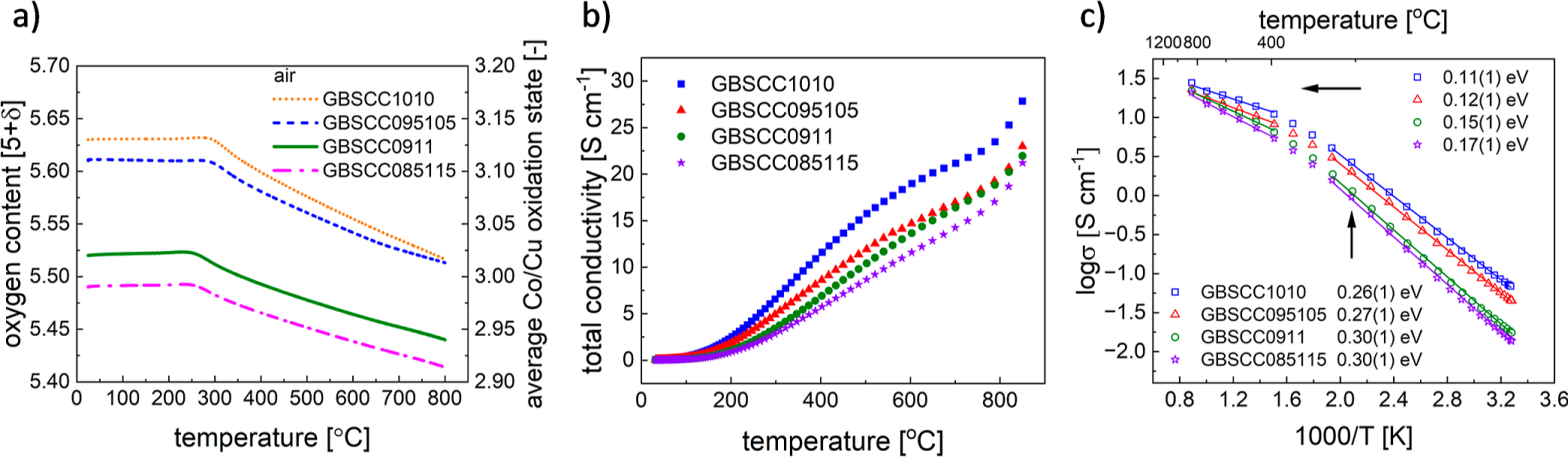
(a) Temperature dependence of the oxygen content and (b) total electrical conductivity for the GBSCC series in air as a function of temperature and (c) conductivity in Arrhenius coordinates.

In order to analyze changes in the oxygen content in GBSCC materials with temperature, TG measurements were carried out in different atmospheres. Figure 3a shows the temperature dependence in air, with the starting point taken on the basis of iodometric titration results. All the compositions demonstrate a similar oxygen-releasing route, which starts at around 250–300 °C and shows a tendency for the onset of the oxygen release to be at lower temperatures for compounds with higher Cu-doping content. In Figure S5a–c, the comparable behavior occurring in other atmospheres is presented (i.e., pure O_2_, 1 vol. % O_2_ in Ar, and 5 vol. % H_2_ in Ar). As expected, the materials show higher total oxygen content in atmospheres with higher pO_2_. Also, the (additional) oxygen vacancy-generating process occurs in all atmospheres. Finally, in the reducing 5 vol. % H_2_ gas, the multistep decomposition proceeds at above 200–250 °C, with the final products (>500 °C) being mixtures of metallic and oxide phases (as derived from XRD studies).

Results of the total electrical conductivity (σ) in the air of GBSCC materials are exhibited in Figure 3b. With the temperature rising, the σ values grow significantly for all compositions, denoting an activated conduction mechanism. Moreover, conductivity in the series decreases for higher Cu-doping levels at the same temperature, which can be explained due to the respective lower oxygen content, affecting the “–O–Cu–O–” and “–O–Co–O–” charge transfer.^52^ Also, since the lower and high-temperature activation energies, *E*_a_ (Figure 3c), increase with *x*, it can be derived, by analogy to data for Co- and Mn-based perovskites,^54,55^ that the charge transfer involving cobalt cations is preferable. Nevertheless, the *E*_a_ values remain moderately low for all samples, and the σ values are still higher than 10 S cm^–1^ in the range of 600–800 °C, indicating favorable transport characteristics. With the typical relative density of the measured sinters of 85–88% (Figure S6a,b), the porosity-corrected values are also somewhat higher, as should be multiplied by a 1.2–1.3 factor (as derived by using the Bruggeman effective medium theory^56^).

### Results of DFT Calculations in the GdBa_1–*y*_Sr_*y*_Co_2–*x*_Cu_*x*_O_5+δ_ System

**Figure 4:**
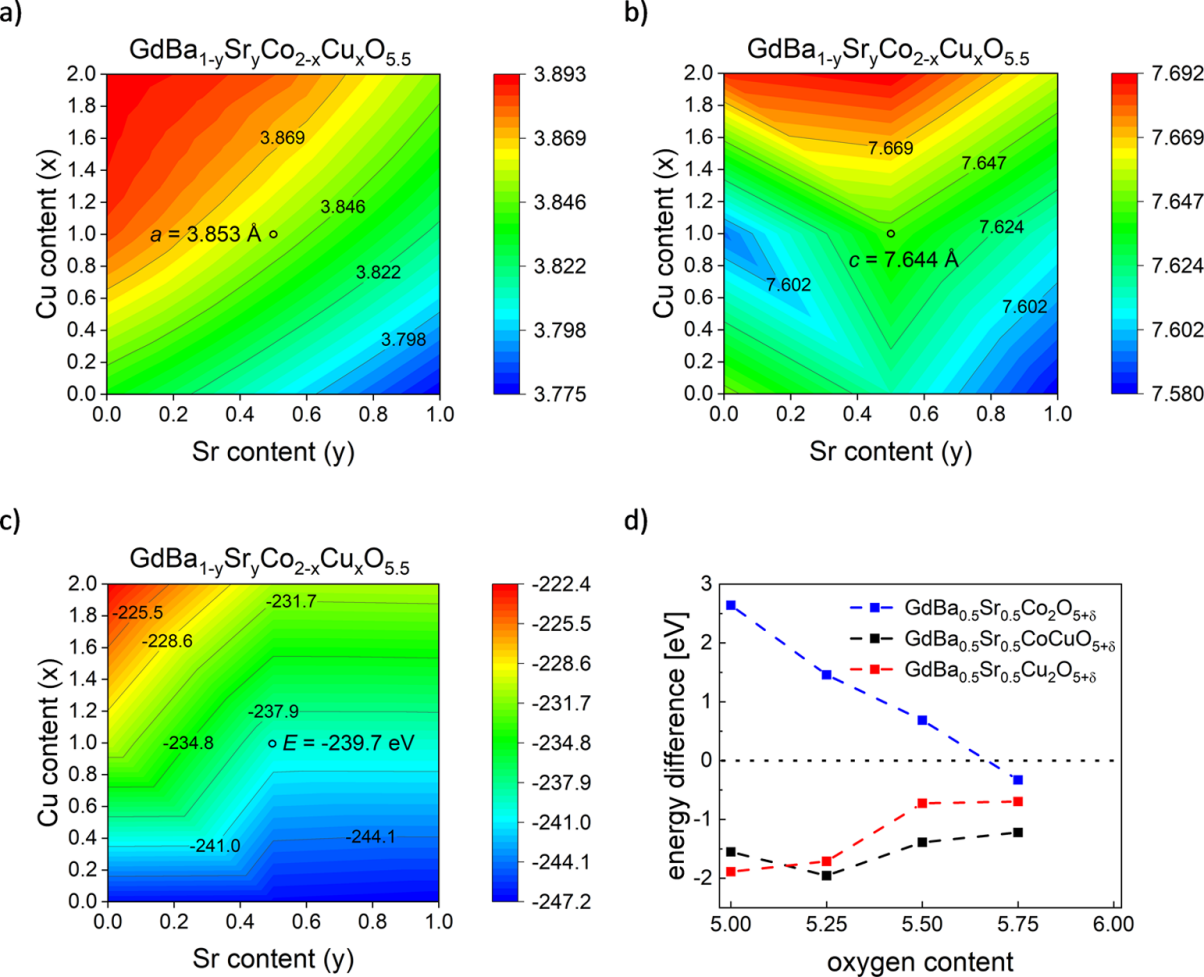
DFT-derived unit cell parameters: (a) *a* and (b) *c* as a function of Sr and Cu content in GdBa_1–*y*_Sr_*y*_Co_2––*x*_Cu_*x*_O_5.5_, (c) calculated total VASP energy (eV for the unit cell), and (d) calculated energy difference between GdBa_1–*x*_Sr_*x*_Co_2–*y*_Cu_*y*_O_6_ and materials with different oxygen content, with the energy of the released oxygen taken into account. The minimum represents the most stable configuration. Please note that the same structural model was used for all calculations, despite the fact that Cu-rich compositions could not be obtained as phase-pure.

Since the experimental results (see TG results and iodometric titration data) show that the oxygen content in the examined compounds varies between 5.49 and 5.64, the presented calculations are shown for a relatively simple case of δ = 0.5 (the oxygen content is equal to 5.5). The reported data are presented in 2D color contour plots, in which the axes correspond to the strontium and copper content, while the *z*-axis values are shown using different colors. Red and blue colors denote the highest and lowest values, respectively. The calculated *a* and *c* unit cell parameters are shown in Figure 4a,b. To make it more simple for calculations and easier for comparing, it was assumed that all GdBa_1–*y*_Sr_*y*_Co_2–*x*_Cu_*x*_O_5+δ_ compositions in the whole series maintain the same crystal structure and symmetry (and can be successfully obtained).

It can be seen that the *a* unit cell parameter should increase for the higher amount of copper and barium in the GdBa_1–*y*_Sr_*y*_Co_2–*x*_Cu_*x*_O_5.5_ series, while it ought to decrease for the higher strontium and cobalt content. This behavior is expected due to differences in the ionic radii of the elements.^48^ Importantly, the derived DFT value of *a* = 3.853 Å for GdBa_0.5_Sr_0.5_CoCuO_5.5_ is in good agreement with the experimental value for GBSCC1010, which is equal to 3.8625(1) Å. The expected increasing trend is also observed in the measured materials (Table S1). Interestingly, for the calculated unit cell parameter *c*, the DFT picture shows more complex characteristics (Figure 4b). Apart from the right-side bottom minimum, the lowest values are also anticipated for Ba-rich samples, in which the cobalt and copper amounts are similar. The calculated *c* parameter of 7.644 Å for GdBa_0.5_Sr_0.5_CoCuO_5.5_ is larger than the measured value for GBSCC1010, which is equal to 7.5809(1) Å. Nevertheless, taking into account the simplicity of the used model, it can be considered reasonable. Comparing the expected and obtained results, it should be taken into account that samples with high copper content could not be synthesized as a single phase. This can also be influenced by the changing oxygen content in the main phase, as well as the discussed possibility of different arrangements of Gd-related oxygen ions in the lattice (Figure S3). Overall, the adopted DFT methodology seems to yield acceptable results if compared with other reported data (Table S3). As can be seen from Figure S7, the expected tendency for changes in the unit cell volume in GdBa_1–*y*_Sr_*y*_Co_2–*x*_Cu_*x*_O_5.5_ materials should be similar to that of the *a* parameter. This is also reflected in the studied GBSCC series (Table S1).

The utilized DFT models allowed for obtaining total VASP energies in the GdBa_1–*y*_Sr_*y*_Co_2–*x*_Cu_*x*_O_5.5_ system. As presented in Figure 4c, in the energy contour plot, the lowest energies are obtained for the considered materials with different barium/strontium and cobalt/copper contents. It appears that high Ba-content and Cu-content oxides would not be as stable, but this may be significantly altered if the different oxygen content is considered. In fact, a more detailed discussion about stability as a function of the oxygen amount can also be done.

Usually, eq 1 (see Experimental Section) is used to evaluate the energy related to the oxygen vacancy formation, with negative values indicating that such a process is energetically favorable.^57^ However, it can also be adapted to predict certain tendencies for a series of oxides regarding their oxygen content. As depicted in Figure 4d, in the Cu-free GdBa_0.5_Sr_0.5_Co_2_O_5+δ_ series, the creation of a large number of vacancies should not be energetically favorable (the energy difference is positive). However, there is a minimum present, likely near δ = 0.75. This corresponds to the fact that the formation of the oxygen vacancies should be favorable, but their equilibrated concentration is not expected to be very high. In the case of GdBa_0.5_Sr_0.5_CoCuO_5+δ_, the minimum is at the much lower total oxygen content, while for GdBa_0.5_Sr_0.5_Cu_2_O_5+δ_ (which, however, could not be synthesized), it moves to δ = 0. The resulting trend of decreasing δ for higher *x* values is observed as expected (Table S2), although the δ values are not well predicted.

The performed DFT computations were also used to assess the density of states (DOS) diagrams for the considered materials. As expected, e.g., for the GdBa_0.5_Sr_0.5_CoCuO_5.5_ and GdBa_0.5_Sr_0.5_CoCuO_5.75_ models, the DOS near Fermi level (E_F_) mainly originates from Co and Cu 3d-states, as well as a (smaller) contribution from O 2p-states. Since no energy gap was present near E_F_ in the results, the measured activated behavior of the electrical conductivity (Figure 3c) could be rather linked with the hopping energy barrier.

### Chemical Stability of GdBa_0.5_Sr_0.5_Co_2–*x*_Cu_*x*_O_5+δ_ with Selected Solid Electrolytes

**Figure 5:**
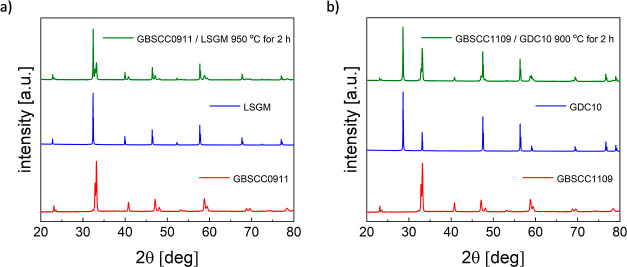
Results of XRD studies for the annealed mixtures of GBSCC0911 and selected solid electrolytes. (a) Data for GBSCC0911 and LSGM and (b) data for GBSCC0911 and GDC.

When it comes to the evaluation of chemical stability with typical solid electrolyte materials, La_0.8_Sr_0.2_Ga_0.8_Mg_0.2_O_3−δ_ and Ce_0.9_Gd_0.1_O_3−δ_ were selected for the studies, as these oxides were further used in the characterization of the electrochemical properties of GBSCC-based electrodes.^58,59^ The stability measurements were done by mixing the respective GBSCC compounds with LSGM or GDC powder with a 1:1 weight ratio and annealing at 950 or 900 °C in air. As shown in Figure 5a,b, the XRD results show no impurities or secondary phases detected after annealing of GBSCC0911 with the electrolytes. Similarly, GBSCC1010 demonstrated the same stability, as presented in Figure S8a,b. Also, the remaining compounds were tested in a similar way, proving their compatibility with both solid electrolytes (not shown in this paper). Thus, it is sufficiently proven that both LSGM and GDC can be assigned as electrolyte supports not only in symmetrical cells but also for full cell manufacturing. However, long-term data for the LSGM electrolyte indicated ongoing strontium segregation at the interface, as indicated below.

### Electrochemical Properties of the GBSCC-Based Oxygen Electrodes

As previously reported for the Cu-based La_1.5_Ba_1.5_Cu_3_O_7±δ_ electrodes, the presence of copper enables to manufacture electrode layers at temperatures as low as 750 °C.^60^ In the case of the considered GBSCC oxides with a Co/Cu ratio close to 1, effective sintering can still be done at a relatively low temperature of 900 °C. Obviously, it is advantageous in comparison to state-of-the-art electrode materials.

**Figure 6:**
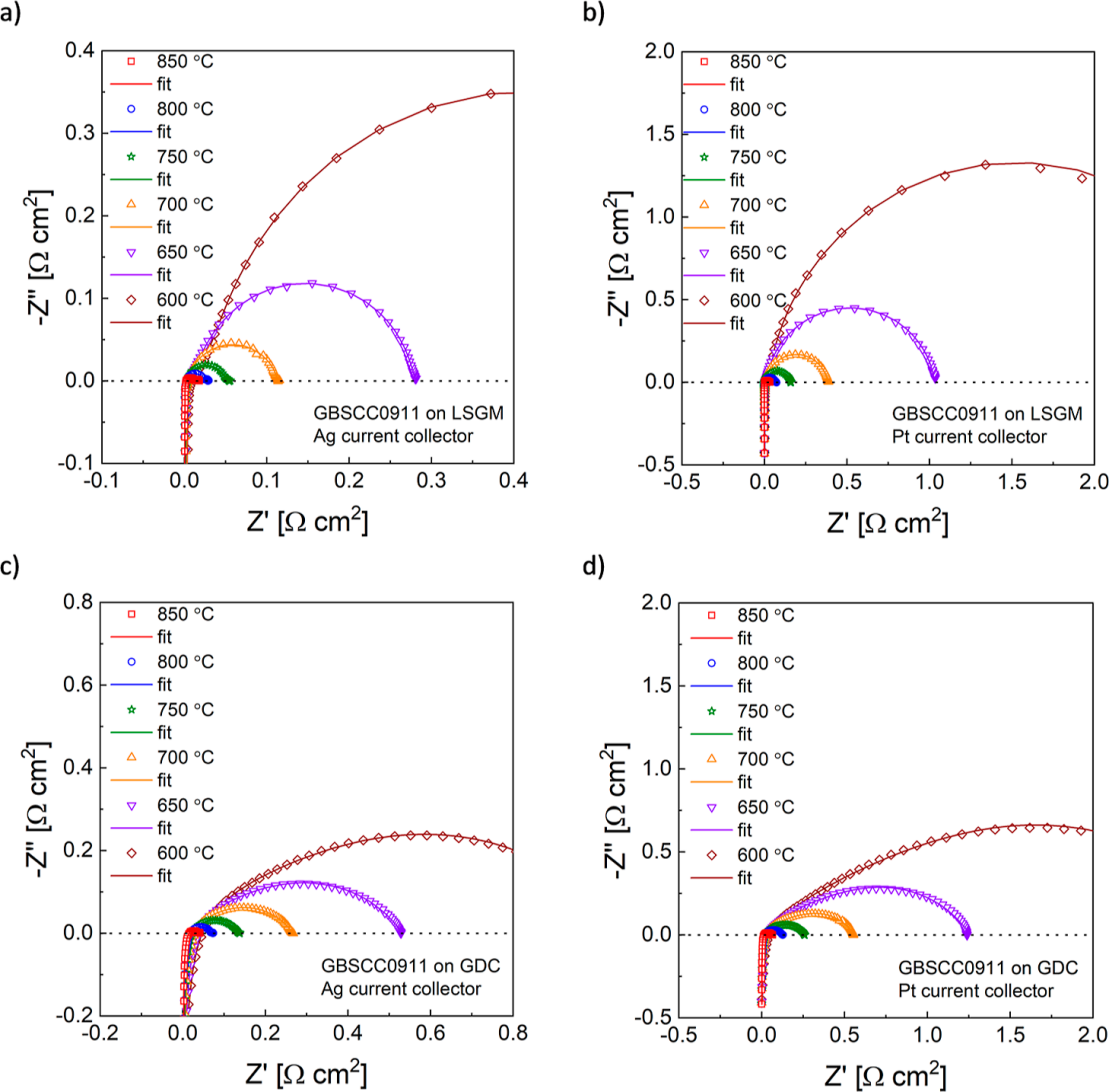
EIS data for the symmetrical cells with GBSCC0911 electrodes: (a) LSGM-based with Ag, (b) LSGM-based with Pt, (c) GDC-based with Ag, and (d) GDC-based with Pt. Data recorded in the 600–850 °C temperature range. Respective fits are also shown.

**Figure 7:**
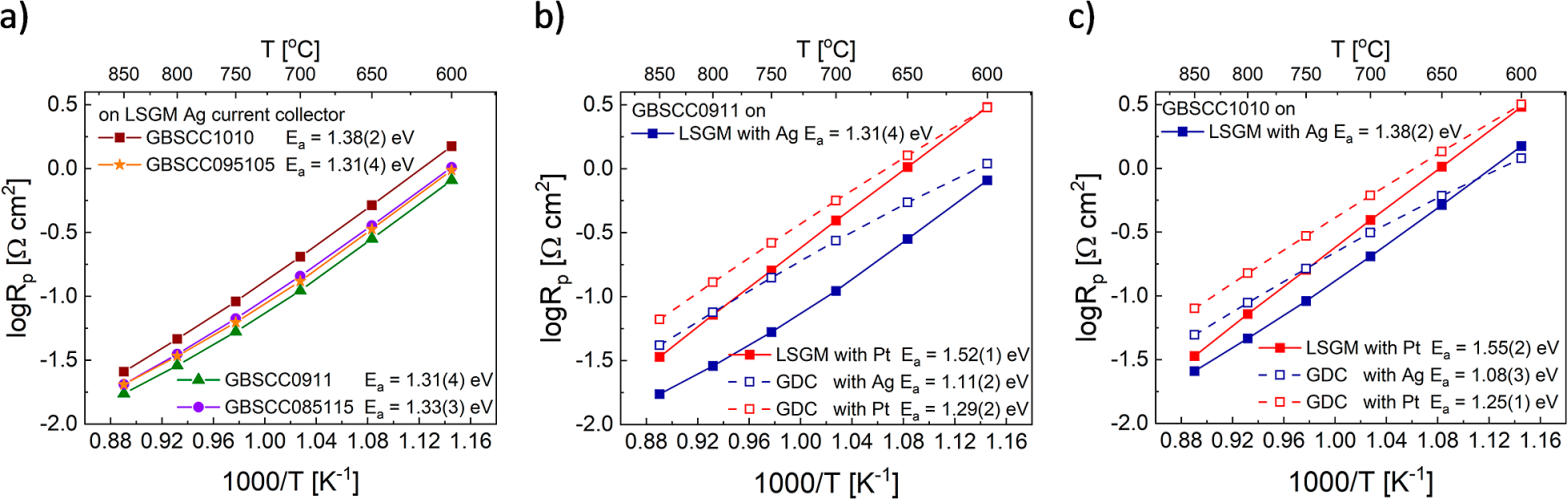
Electrode polarization resistance *R*_p_ presented in Arrhenius-type coordinates for (a) selected GBSCC compositions, (b) GBSCC0911, and (c) GBSCC1010 on the LSGM/GDC electrolyte with the Ag/Pt current collector.

Symmetrical cells that were used for electrochemical measurements were manufactured on LSGM or GDC solid electrolyte pellets with a thickness of ca. 300 μm, and an Ag or Pt current collector was used. As shown in Figure 6a, a clear temperature dependence of EIS arcs can be seen for the electrode layer of GBSCC0911 based on the LSGM electrolyte and with Ag. The oxygen electrode reaches a minimum polarization resistance (*R*_p_) value of 0.017 Ω cm^2^ at 850 °C. For another tested LSGM-supported symmetrical cell with a GBSCC1010 electrode layer, the lowest recorded value of *R*_p_ is 0.026 Ω cm^2^ at 850 °C (Figure S9a). The lower *R*_p_ of the GBSCC0911 electrode indicates enhanced electrocatalytic activity for the material with a higher Cu content, as also observed for other testing temperatures (600–800 °C). However, there is no linear trend in the whole series of the tested materials (Figure 7a), as the values for GBSCC085115 are higher than those for the GBSCC0911 oxygen electrode. This may originate from the fact that this is the final composition with a still negligible amount of secondary phases. Overall, it can be stated that the obtained *R*_p_ values are similar or even lower in comparison with those for Co-based double-perovskite materials,^30,61,62^ implying a competitive advantage of the studied Cu-doping in the GBSCC series.

Cells based on the LSGM electrolyte (with the same thickness), in which a Pt current collector was used (Figures 6b and S9b), showed significantly worsened characteristics at lower temperatures; however, the relative change was found to decrease at higher temperatures. This is clearly observed in Figure 7b,c, in which the higher activation energy of the temperature changes of *R*_p_ is evident (the increase is ca. 0.2 eV). This result indicates the catalytic role of Ag at lower temperatures, which is known from the literature.^63^

Surprisingly, if the electrolyte was GDC, much higher electrode polarization resistance was registered, both for GBSCC0911 (Figure 6c) and GBSCC1010 (Figure S9c) electrodes. The behavior was still worsened (in the same manner as for LSGM-based cells) if the Pt current collector was selected, as shown in Figures 6d and S9d. Overall, analyzing the activation energy of *R*_p_ it can be noticed that the values are generally lower for the GDC-based cells, and several other trends can be observed. For example, replacing Ag with Pt results in an increase of *E*_a_ by ca. 0.2 eV for both solid electrolytes and both electrodes. Also, the GBSCC0911 electrode shows somewhat smaller activation energy values for both LSGM-based cells, but this is reversed for GDC-based ones. Significant differences in the behavior as well as a relatively large discrepancy of the *R*_p_ values between cells utilizing LSGM and GDC electrolyte supports and having Pt or Ag current collectors prompted more detailed studies on the origin of the observed characteristics. The research in this matter involved SEM studies as well as DRT analyses.

The SEM micrographs (Figure S10a) of the electrode–electrolyte interface for the symmetrical cell with the GBSCC0911 oxygen electrode show very good adhesion between the electrode layer and the LSGM electrolyte after the short-term electrochemical measurements. This can be related to the suitable TEC value of GBSCC0911, which does not differ significantly from the electrolyte, as well as the fact that both materials exhibit a perovskite-type structure. Additionally, sufficient porosity of the electrode layer can be observed, which enables adequate gas adsorption on its surface and diffusion into the electrode. The thickness of the electrode layer is ca. 30 μm. Moreover, as can be derived from the EDS results, there is no elemental segregation between the electrode and electrolyte, indicating good short-term compatibility between GBSCC0911 and LSGM materials. In order to assess the long-term stability of the electrochemical performance of the GBSCC materials, 120 h tests were conducted at 700 °C. As can be seen in Figure S11, the total *R*_p_ of the GBSCC1010 and GBSCC0911 electrodes on the LSGM electrolyte and with the Ag current collector does not stabilize during the measurements. However, the relative increase for the material with a higher Cu content is smaller. Furthermore, SEM results revealed that Sr segregation takes place at the electrode/electrolyte interface for the GBSCC1010 material (Figure S10b), but it is largely suppressed in the case of the GBSCC0911 electrode (Figure S10c). This indicates the additional benefit of choosing a material with a higher Cu content. Importantly, replacement of Ag with Pt current collector also results in improved characteristics, and the values stabilize after ca. 80 h (the relative increase is 0.05% h^–1^ in the last 40 h). It is evident that a constant increase of *R*_p_ for cells with the Ag current collector, also those with the GDC electrolyte, indicates that silver does not warrant good stability. However, decreasing the temperature to 600 °C indeed results in much more stable behavior. There is no strontium segregation at the interface of GBSCC0911 and GDC (Figure S10d), nor is there any unwanted reactivity involving the other elements. In this case, and for the Pt current collector, the polarization resistance increases by only 0.04% h^–1^ in the last 40 h of operation at 700 °C. This magnitude of the *R*_p_ increase is actually typical for different tested electrodes.^64^ Worth mentioning, good performance could also be obtained for the anode-supported design (YSZ electrolyte), in which a GDC buffer layer and a Pt current collector were used.^47^

### DRT Analysis of the Electrochemical Properties of the GBSCC0911 Oxygen Electrode

**Figure 8:**
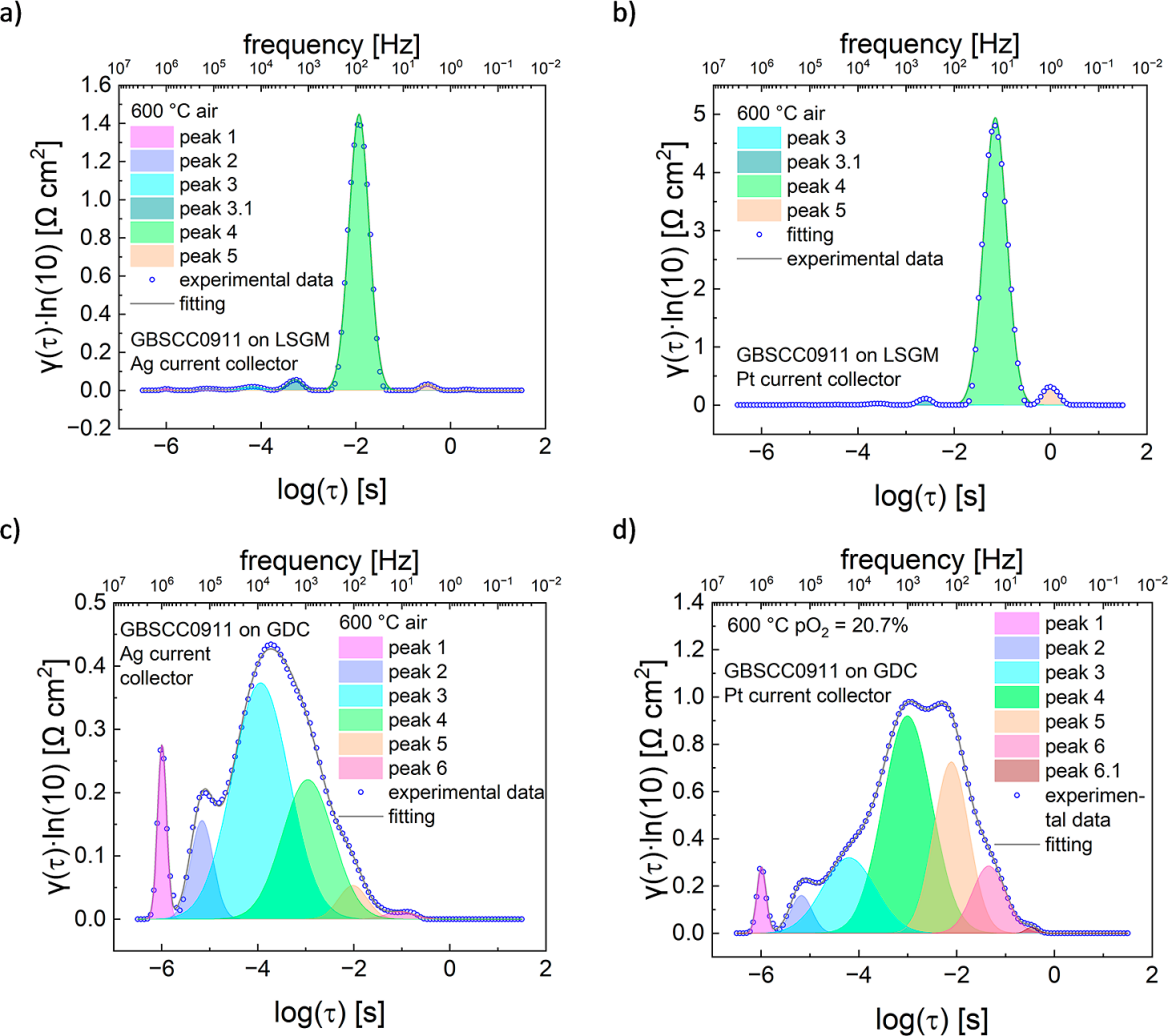
Results of DRT analysis as a function of τ for different symmetrical cells with GBSCC0911 electrodes. Data at 600 °C for the (a) LSGM electrolyte and Ag current collector, (b) LSGM and Pt, (c) GDC and Ag, and (d) GDC and Pt.

**Figure 9:**
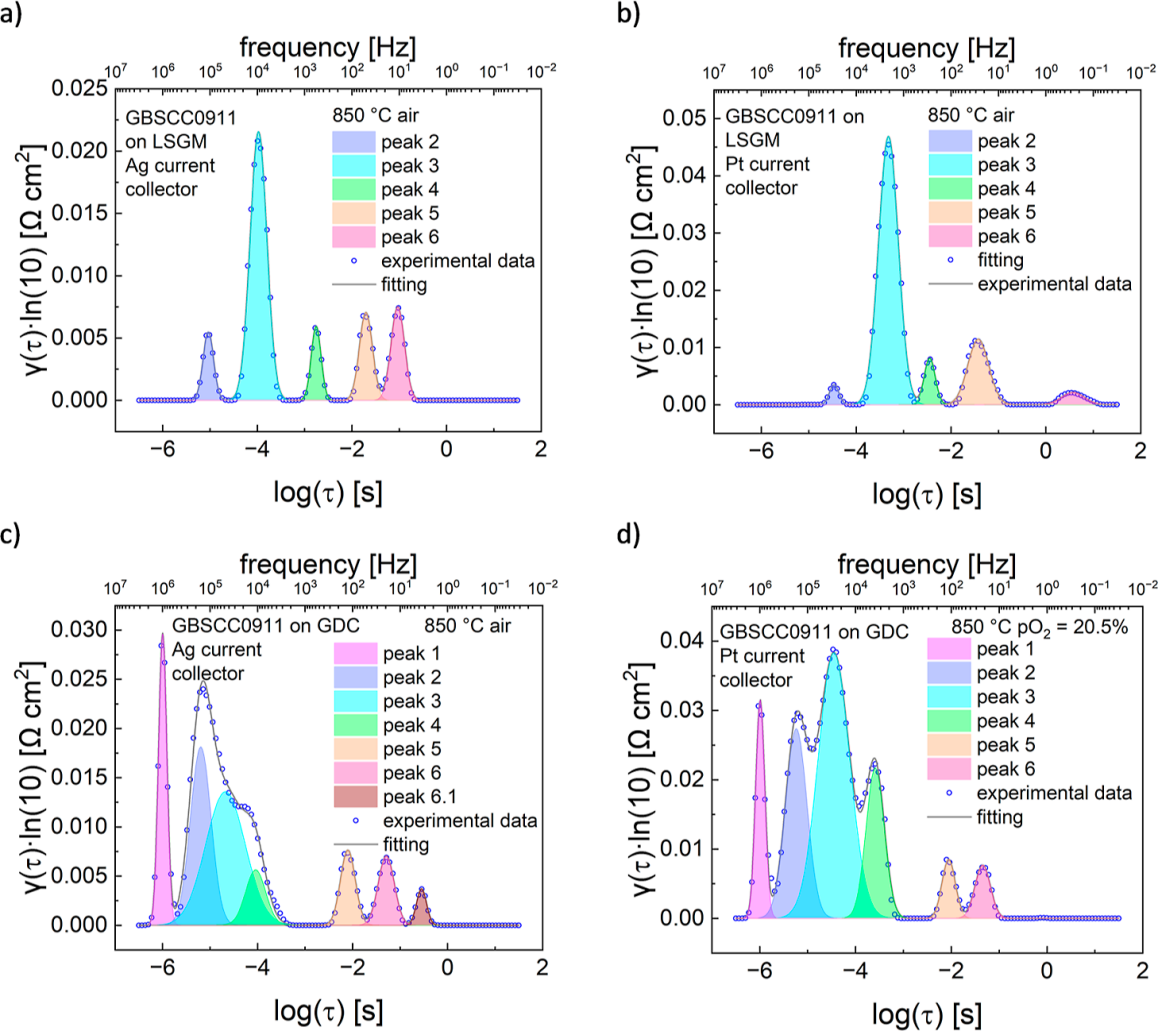
Results of DRT analysis as a function of τ for different symmetrical cells with GBSCC0911 electrodes. Data at 850 °C for the (a) LSGM electrolyte and Ag current collector, (b) LSGM and Pt, (c) GDC and Ag, and (d) GDC and Pt.

The ORR occurring at the MIEC electrode can be divided into six elementary processes, all of which are characterized by the respective reaction order parameters *m*.^65−67^ In brief, the steps are as follows: adsorption of O_2_ (*m*_ad_ = 1), dissociation of O_2_ (*m*_dis_ = 1/2), two-step reduction (*m*_red1_ = 3/8 and *m*_red2_ = 1/8), lattice incorporation of the reduced oxygen (*m*_inc_ = 0), and O^2–^ migration through the electrode/electrolyte interface (*m*_mig_ = 0). Since DRT usually allows us to distinguish processes having different relaxation times (τ), measurements as a function of pO_2_ at different temperatures bring valuable information. For these tests, the EIS data were recorded for GBSCC0911 electrodes on LSGM and GDC pellets with both Ag or Pt current collectors used, as presented respectively in Figures 8a–d and 9a–d. Additional results were recorded as a function of pO_2_ for the most stable cell configuration, with GBSCC0911 electrodes, GDC electrolyte, and Pt current collector (Figure S12a–d). The data were analyzed from measurements conducted at 600 and 850 °C.

**Figure 10:**
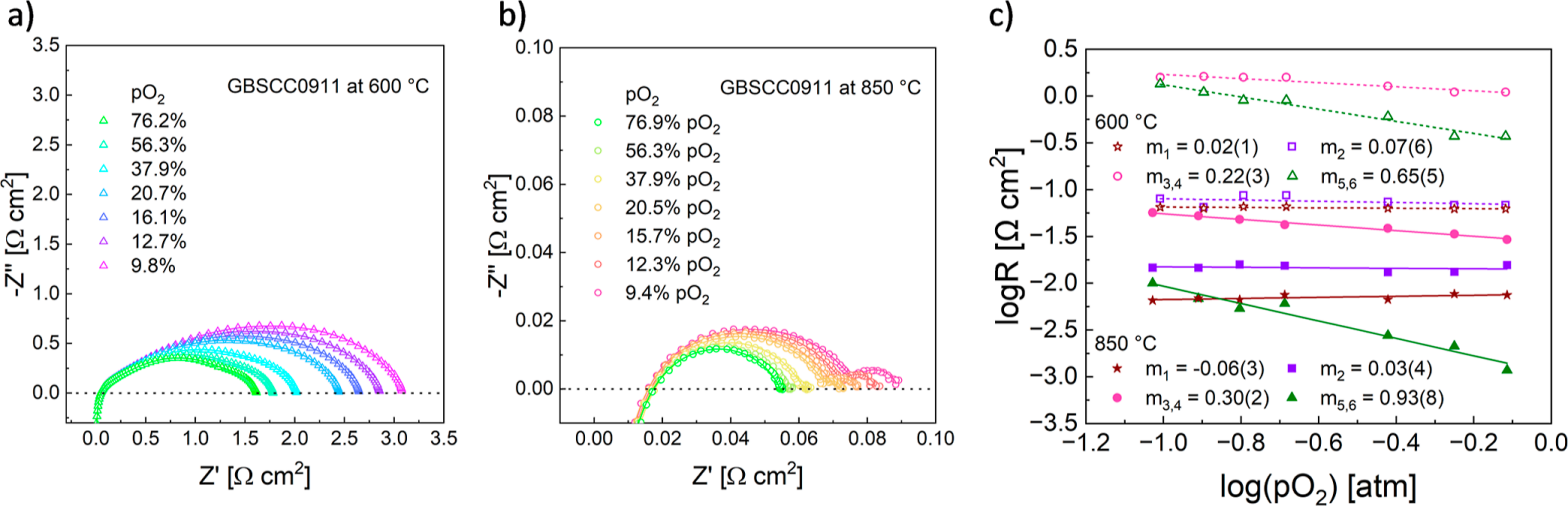
Measured EIS spectra for the Pt,GBSCC0911/GDC/GBSCC0911,Pt cell as a function of pO_2_ at (a) 600 and (b) 850 °C. The lines represent standard fits with the equivalent circuit. (c) Dependence of the derived partial resistances as a function of pO_2_ in log–log coordinates. Note the included slope values *m*, fitted for data registered at 600 and 850 °C.

As can be seen in the presented DRT graphs, not only is the total *R*_p_ different between four different cells (with the same GBSCC0911 electrodes), but importantly, the separated components vary significantly. In order to assess the changes and interpret the origins of the multiple peaks, initially, the pO_2_ dependence was analyzed in more detail. As shown in Figure 10a,b, the recorded EIS curves at 600 °C and 850 °C of the GBSCC0911 electrode demonstrate a continuous trend, with a decrease of the polarization resistance when the atmosphere is changed from ca. 9 to 76% oxygen. All of the obtained DRT characteristics are complex, showing six or more peaks. However, the peaks behave differently as a function of the oxygen partial pressure and can be gathered into four distinctive groups (having different τ as well). Since peak 1 is practically unchanged with pO_2_ (*m*_1, 600 °C_ = 0.02(1), *m*_1, 850 °C_ = −0.06(3), Figure 10c), and considering its low τ (∼10^–6^ s), it can be assigned to the charge transfer process through the electrode/electrolyte interface. For peak 2, it also does not show any significant pO_2_ dependence at both temperatures (*m*_2, 600 °C_ = 0.07(6), *m*_2, 850 °C_ = 0.03(4)), and taking into account its low τ < 10^–5^, it can be ascribed to the reduced oxygen incorporation into the vacancy site of the electrode material crystal lattice, which step also has a theoretical *m* = 0. Screening of the data showed that two peaks present in the 10^–5^ s < τ < 10^–3^ s range evolve with pO_2_ with an (averaged) slope *m*_3,4_ of about 0.25 (*m*_3,4, 600 °C_ = 0.22(3), *m*_3,4, 850 °C_ = 0.30(2), Figure 10c). While it was not possible to reliably show their separated dependences, the combined behavior strongly indicates that they correspond to the reduction processes, which, considered as a single step, exhibit a theoretical *m* = 0.25. The integrated area of both these peaks dominates the total resistance, which indicates that this step is the hindering part of the ORR taking place at the GBSCC0911 electrode. Nevertheless, at 600 °C and lower pO_2_, the dominance of this step is not as strong as at 850 °C. Also, at 850 °C and lower oxygen partial pressures, there is a right-side shoulder visible on peak 4 (Figure S12c), which was added in the calculations with peaks 3 and 4. The combined remaining peaks (two or three), which are present for τ > 10^–3^ s show a slope of *m*_5,6, 600 °C_ = 0.65(5) and *m*_5,6, 850 °C_ = 0.93(8). This indicates that the dissociation process at the lower temperature and the adsorption step at 850 °C contribute mainly to that part of the polarization resistance. At 850 °C, this contribution, however, is the smallest of all, except for the lowest pO_2_. Overall, the behavior is comparable to that reported for other highly active oxygen electrodes.^68,69^ At the same time, more complex spectra with numerous peaks allow us to speculate that both, Co- and Cu-related surface sites, as well as both ions present in the bulk, contribute to the respective surface- and bulk-related steps of the ORR. This, however, should be confirmed in further studies.

With the identified contribution of different steps of the ORR, it is possible to present a deeper comparison of the cells differing in the electrolyte and current collector. The relevant data for the partial polarization resistance values are included in Table S4. The respective components were recognized on the basis of τ vales and their changes with temperature. It is evident that the charge transfer through the electrode/electrolyte interface is much easier for LSGM/GBSCC0911 (data from 850 °C are practically close to zero). At 600 °C, for GDC-based cells, this contribution is not very important to the total *R*_p_, while at 850 °C, it constitutes a larger but still not dominant part of the polarization resistance. Importantly, the values for both cells with GDC are comparable, supporting a proper identification of this contribution. Structurally, the easier transfer may be related to a better structural correspondence in the case of both perovskite-type phases present. For the reduced oxygen incorporation into the vacancy site step, again, for the LSGM-based cells, the values are significantly lower at both temperatures. It may be initially surprising; however, a plausible interpretation can be provided that this result indicates that the process occurs effectively in the vicinity of the TPB, i.e., near the interface with the electrolyte support. Otherwise, it would be very hard to explain the influence of the electrolyte on this particular step of ORR. It is evident that the presence of Ag enhances catalytic activity toward the oxygen reduction steps, especially at a lower temperature, as *R*_3,4_ is significantly lower for the respective cells with the silver current collector (despite the type of the electrolyte). This confirms the catalytic influence of silver.^63^ Also, it is crucial to mention that it is the largest component of the total *R*_p_ for all cells at both temperatures. It can also be speculated (by comparison of peaks 3 and 4 for both cells with the LSGM electrolyte) that at lower temperatures, the first step of the oxygen atom reduction process is more limiting, while at higher temperatures, it is the second step. However, this needs further confirmation. Regarding the last component, *R*_5,6_, it is much smaller if Ag was used, also showing that silver facilitates the oxygen molecule dissociation and adsorption processes, again, especially at the lower temperature. Such influence is less visible at 850 °C, but it is still noteworthy.

### Electrochemical Performance of the Optimized Full Cells with GBSCC-Based Oxygen Electrodes

**Figure 11:**
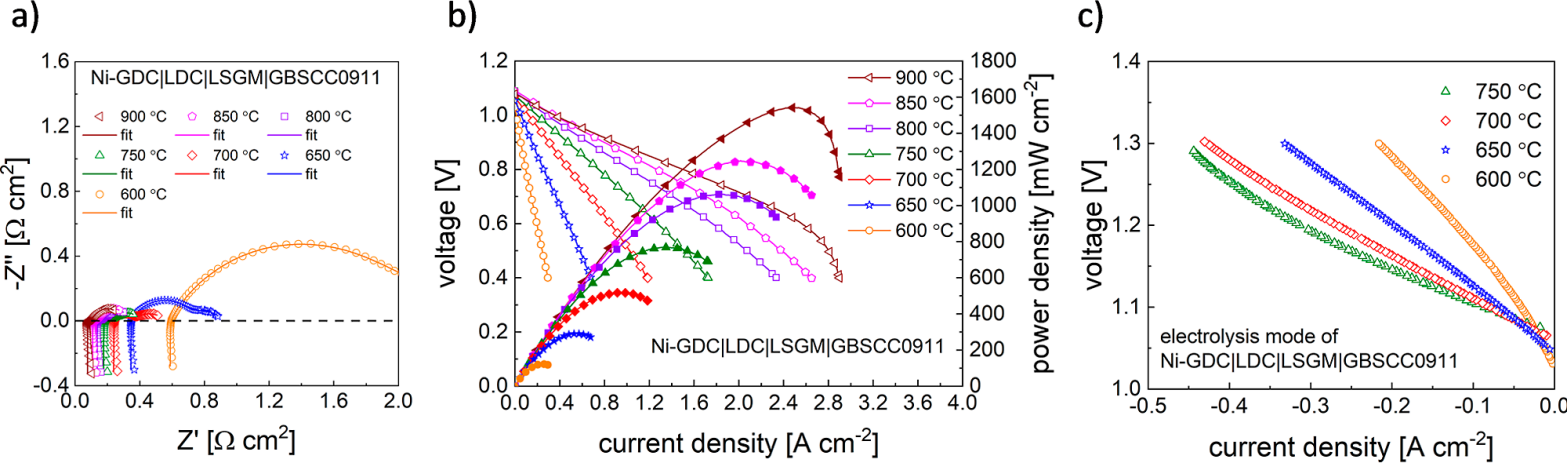
(a) EIS data measured under open circuit conditions, (b) voltage–current density (*V*–*I*) curves and power density output, and (c) performance in the electrolyzer mode for the Ni-GDC|LDC|LSGM|GBSCC0911 single cell. V–I curves measured for the humidified H_2_ flow of 50 mL min^–1^.

In order to have a comprehensive evaluation of the oxygen electrode performance regarding the power output, button-type single cells were manufactured with Ni-GDC cermet as the anode, an LDC buffer layer at the anode side, a thin LSGM electrolyte pellet (ca. 200 μm thickness), and either GBSCC0911 or GBSCC1010 as the oxygen electrode. As shown in Figure 11a, the EIS curves demonstrate low polarization resistance values as well as low ohmic resistance in the whole measured temperature range. Specifically, three arcs are easily detected throughout the range of 600–900 °C, with the arc in the high-frequency range becoming more and more overlapping with the middle-frequency one at lower temperatures. Generally, the same behavior was observed for the cell with GBSCC1010 as the oxygen electrode (Figure S13a). Overall, the characteristics are in agreement with the above-discussed results of DRT studies.

In Figure 11b, the presented voltage–current curves show an increasing maximum specific current density with rising temperature. For example, the Ni-GDC/LDC/LSGM/GBSCC0911 cell reaches a power output above 1500 mW cm^–2^ at 900 °C and above 1240 mW cm^–2^ at 850 °C, which is similar to the single cell of Ni-GDC/LDC/LSGM/GBSCC1010, presented in Figure S13b. Those exceptionally high output performance values indicate that both oxygen electrode materials are indeed very promising. Also, the decreased cobalt content in GBSCC0911 makes it more competitive, especially in comparison to those based on high Co-content compositions.^28,34,70^ For temperatures equal to or lower than 700 °C, the GBSCC0911-based cell shows improved characteristics, e.g., about a 5% power density increase at 700 °C. Overall, the reported performance is much better in comparison to cells with PrBaCo_2_O_5+δ_,^17^ NdBaCo_2_O_5+δ_, or GdBaCo_2_O_5+δ_ electrodes.^23^ In the electrolysis mode, both cells perform very well (Figures 11c and S13c). For example, the Ni-GDC/LDC/LSGM/GBSCC0911 cell reaches 0.44 A cm^–2^ with a voltage of 1.3 V at 750 °C, which indicates that the GBSCC oxygen electrode can also perform effectively in the SOEC mode (i.e., generating oxygen). Also, a higher steam content at the Ni-GDC is expected to further improve these characteristics.

The above results must be understood as proof of the high electrocatalytic activity of the GBSCC materials, although issues related to maintaining their performance over a prolonged period of time must still be resolved.

## Conclusions

High-performance double perovskite-type oxygen electrode materials with the GdBa_0.5_Sr_0.5_Co_2–*x*_Cu_*x*_O_5+δ_ formula and a Co/Cu content close to 1 were successfully developed. The comprehensive measurements performed allowed for assessing the physicochemical properties of the oxides, with details about crystal structure, oxygen content, thermal expansion, electrical conductivity, and thermal and chemical stability provided. Compositions with 1 ≤ *x* ≤ 1.15 were found to be especially interesting, and among them, GdBa_0.5_Sr_0.5_Co_0.9_Cu_1.1_O_5+δ_ composition, with reduced Co content, was documented to be the best candidate oxygen electrode material. Studies of the electrocatalytic activity with the use of DRT analyses allowed distinguishing elementary steps of the electrochemical reaction. It could be concluded that at 600 and 850 °C, the limiting step of the ORR is the oxygen atom reduction process. The developed GBSCC0911 electrode displayed excellent electrocatalytic activity with low electrode polarization resistance values, e.g., 0.017 Ω cm^2^ at 850 °C, 0.029 Ω cm^2^ at 800 °C, and 0.111 Ω cm^2^ at 700 °C. Furthermore, the role of the current collectors (Ag and Pt) was evaluated. Apparently, silver can facilitate the ORR; however, platinum allows for obtaining a stable behavior in long-term measurements. Also, there is Sr segregation occurring at the interface with the LSGM electrolyte for the GBSCC1010 electrode, but this can be suppressed if the optimized GBSCC0911 composition is used. For the laboratory-scale LSGM-supported cells, high power outputs exceeding 1240 mW cm^–2^ at 850 °C and 1060 mW cm^–2^ at 800 °C could be obtained. Despite the reduced cobalt content in the electrode, the GdBa_0.5_Sr_0.5_Co_0.9_Cu_1.1_O_5+δ_-based cell showed improved characteristics over the GdBa_0.5_Sr_0.5_CoCuO_5+δ_-based one at lower temperatures, ≤700 °C. The electrolysis mode of operation was also tested, with a current density of about 0.43 A cm^–2^ recorded for 1.3 V at 700 °C.

## Supporting Information Available

A model of a GdBa_0.5_Sr_0.5_CoCuO_5.5_ supercell with the presented method of cobalt substitution by copper; XRD data for GBSCC samples with *x* = 1.2–2; simplified models of different oxygen arrangements in the crystal structure; refined structural parameters of studied materials; HT-XRD data with cell parameters dependence on temperature and calculated TEC values for GBSCC1010 and GBSCC095105; oxygen content in GBSCC samples at RT; temperature dependence of the oxygen content in the selected GBSCC in different atmospheres; SEM micrographs of the GBSCC0911 pellet; DFT calculations and experimental structural data for different GBSCC materials; DFT-derived unit cell volume in GdBa_1–*y*_Sr_*y*_Co_2–*x*_Cu_*x*_O_5.5_; XRD studies at RT for the annealed mixtures of GBSCC1010\LSGM and GBSCC1010\GDC; EIS data for the symmetrical cells with GBSCC1010 electrodes and different electrolytes and current collectors; morphology with EDS maps for different tested GBSCC0911 electrode/electrolyte interfaces; polarization resistance changes with time for different tested cells; DRT analyses for the selected pO_2_ values for the symmetrical cell with GBSCC0911 electrode and Pt current collector; comparison of components of the total polarization resistance of symmetrical cells with GBSCC0911 electrodes and different electrolytes and current collectors; and EIS data, voltage–current density, power density curves, and performance in the electrolyzer mode for the Ni-GDC|LDC|LSGM|GBSCC1010 single cell (PDF)
