# Supplementary material for: Unveiling the Electrocatalytic Activity of the GdBa0.5Sr0.5Co2–xCuxO5+δ (x ≥ 1) Oxygen Electrodes for Solid Oxide Cells
Source: ACS Appl Mater Interfaces. 2023 Aug 9;15(33):39578–93. doi: 10.1021/acsami.3c08667 (PMC10450687; doi:10.1021/acsami.3c08667)
Supplement: Supplementary file 1 — am3c08667_si_001.pdf [file am3c08667_si_001.pdf]

Supporting Information

Unveiling the electrocatalytic activity of the $GdBa_{0.5}Sr_{0.5}Co_{2-x}Cu_xO_{5+\delta}$ ($x \geq 1$) oxygen electrodes for Solid Oxide Cells

Keyun Li[a], Konrad Świerczek[a,b,*], Piotr Winiarz[a], Agnieszka Brzoza-Kos[a], Anna Stępień[a], Zhihong Du[c], Yang Zhang[d], Kun Zheng[a,b], Kacper Cichy[a], Anna Niemczyk[e,f], Yevgeniy Naumovich[e,f]

[a]AGH University of Science and Technology, Faculty of Energy and Fuels,
al. A. Mickiewicza 30, 30-059 Krakow, Poland
[b]AGH Centre of Energy, AGH University of Science and Technology,
ul. Czarnowiejska 36, 30-054 Krakow, Poland
[c]School of Materials Science and Engineering, University of Science and Technology Beijing,
Beijing, 100083, China
[d]Key Laboratory of Advanced Fuel Cells and Electrolyzers Technology of Zhejiang Province,
Ningbo Institute of Material Technology and Engineering, Chinese Academy of Sciences,
Ningbo, 315201, China
[e]Center for Hydrogen Technologies (CTH2), Institute of Power Engineering,
ul. Augustowka 36, 02-981 Warsaw, Poland
[f]Institute of Power Engineering,
ul. Mory 8, 01-330 Warsaw, Poland
[*]xi@agh.edu.pl

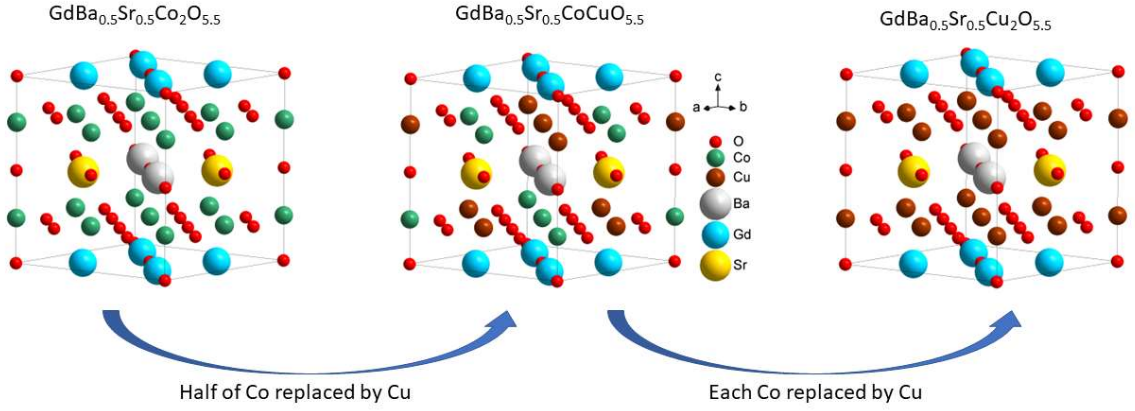

Figure S1. A representative supercell of $GdBa_{0.5}Sr_{0.5}CoCuO_{5.5}$ (middle part). Since the unit cell is doubled along the *x*- and *y*-axis, the exact formula in the unit cell is $Gd_4Ba_2Sr_2Co_2Cu_2O_{22}$. A method of cobalt (green) substitution by copper (brown) is also presented.

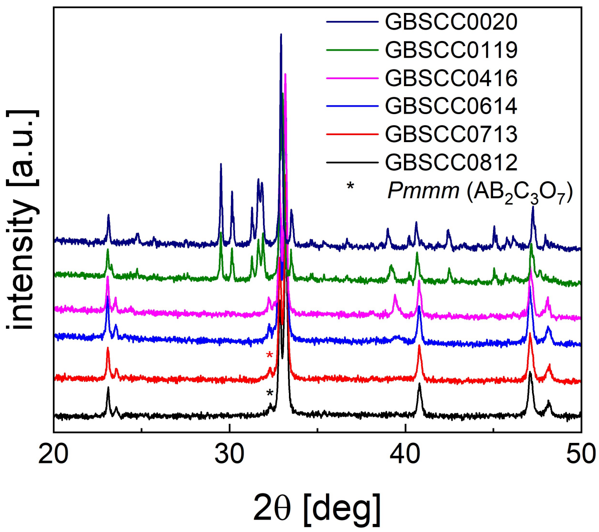

Figure S2. XRD data measured for other GBSCC samples (x = 1.2-2) at RT. The marked secondary phase peak likely originates from the orthorhombic triple perovskite phase. Notice a growing amount of secondary phases, especially if $x \geq 1.9$.

a) **b)**

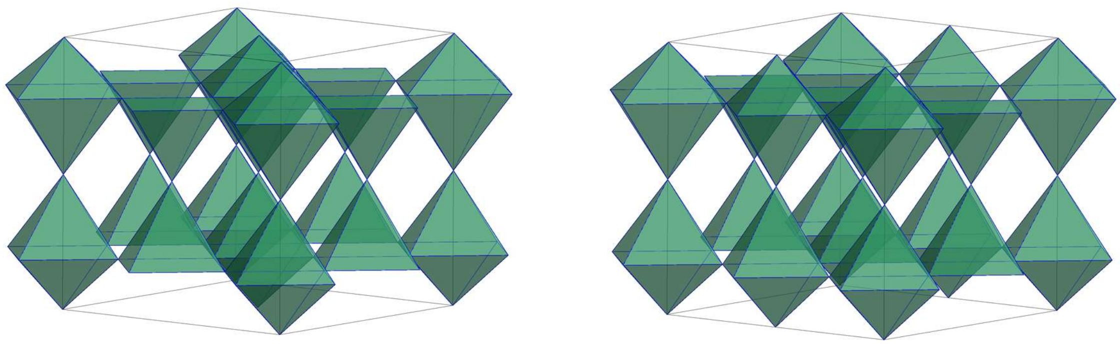

Figure S3. Simplified models of different oxygen arrangements in the *a*-*b* plane associated with Gd, with maintaining $\delta = 0.5$. Only (Co, Cu)O_5 and (Co, Cu)O_6 polyhedra are shown. a) Only octahedra-pyramids connections are present in the cell, b) all, pyramids-pyramids, octahedra-octahedra, and mixed connectivities are observed.

Table S1. The refined structural parameters of the selected GBSCC materials.

composition	unit cell parameter *a* [Å]	unit cell parameter *c* [Å]	unit cell volume *V* [Å^3]	O (0.5,0,*z*) *z* value	R_{wp} [%]
GBSCC1505	3.8566(1)	7.5637(1)	112.50(1)	0.210	1.9
GBSCC1010	3.8625(1)	7.5809(1)	113.10(1)	0.209	2.3
GBSCC095105	3.8629(1)	7.5812(2)	113.13(1)	0.208	2.6
GBSCC0911	3.8633(1)	7.5799(2)	113.13(1)	0.218	2.5
GBSCC085115	3.8640(1)<br>3.8654(1)[1]	7.5784(2)<br>7.5757(2)[1]	113.15(1)<br>113.19(1)[1]	0.215<br>0.217[1]	2.7<br>2.8[1]

[1] The material was sintered twice in the same conditions.

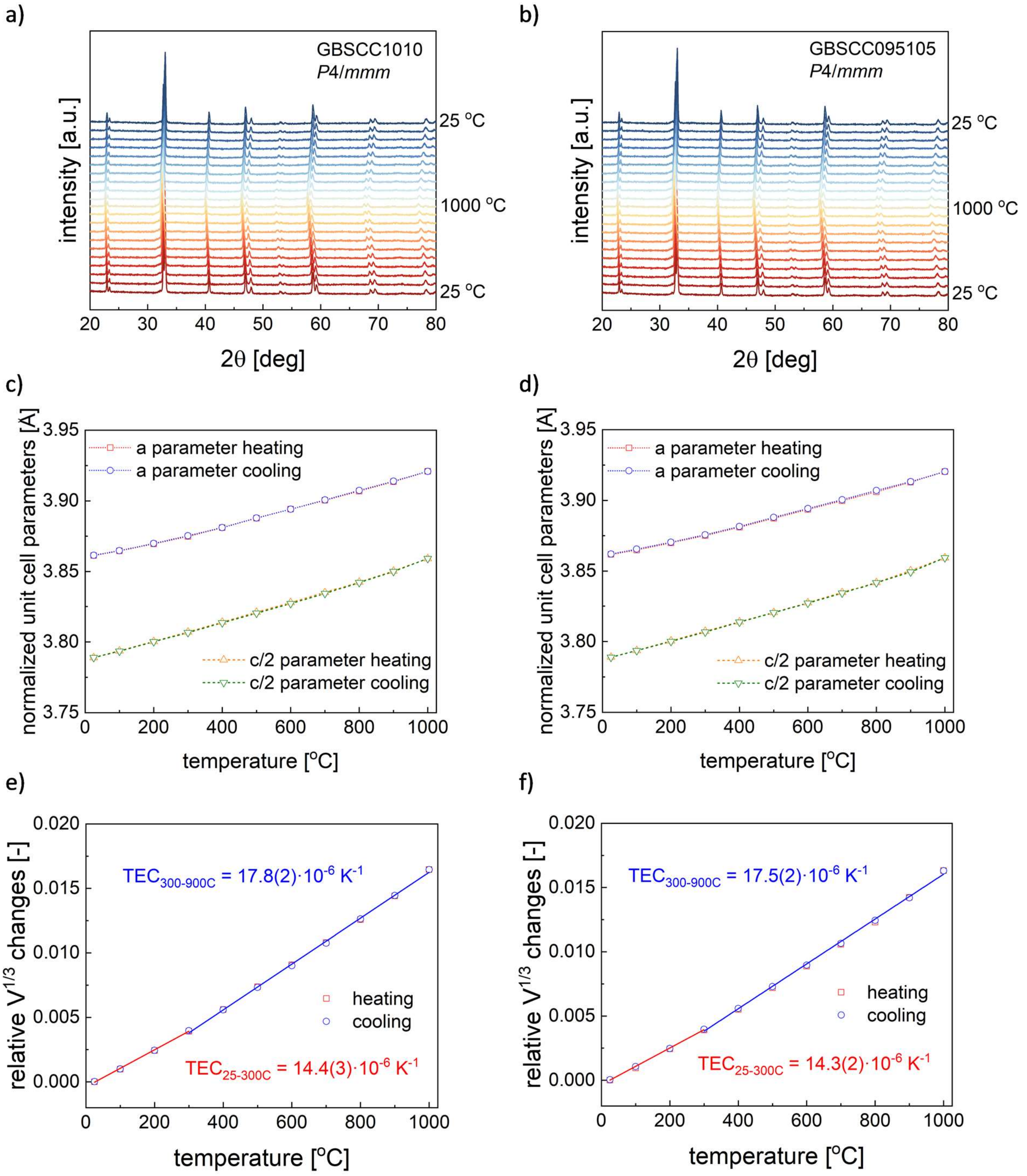

Figure S4. a) HT-XRD data, b) Derived unit cell parameters dependence on temperature, and c) calculated TEC values recorded for the GBSCC1010 material. Similar data are presented for GBSCC095105 in d), e) and f).

Table S2. The evaluated oxygen content in GBSCC samples at RT. Data from the iodometric titration. The error value does not exceed ±0.01.

composition	oxygen content (5+δ)
GBSCC1505	5.59
GBSCC1010	5.64
GBSCC095105	5.61
GBSCC0911	5.52
GBSCC085115	5.49

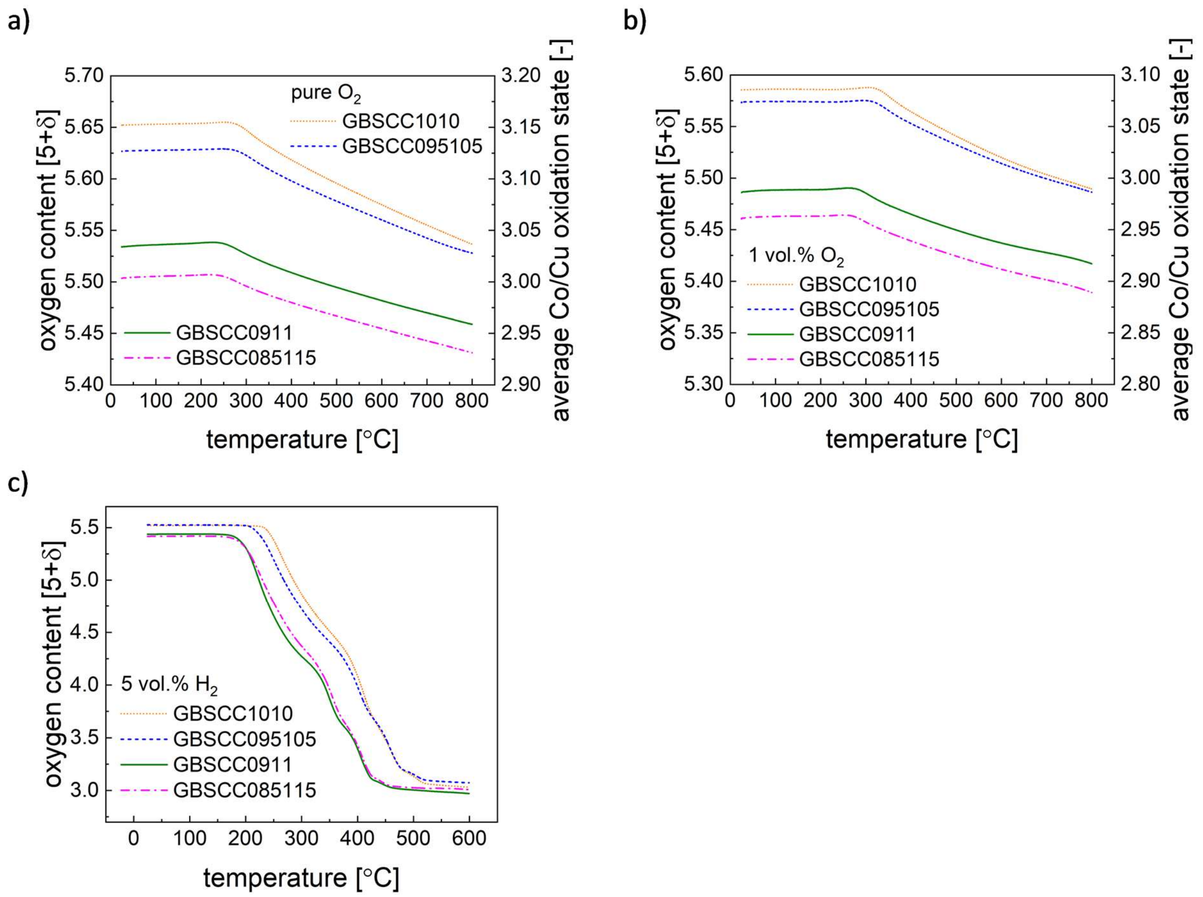

Figure S5. Temperature dependence of the oxygen content in the selected GBSCC materials in: a) oxygen, b) 1 vol.% O_2 in Ar, c) 5 vol.% H_2 in Ar (decomposition curves).

a) b)

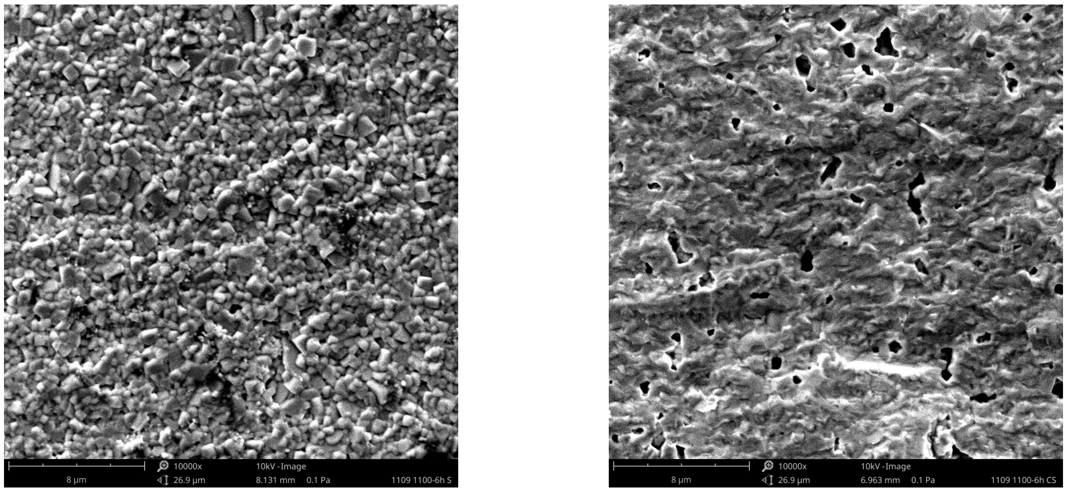

Figure S6. SEM micrographs of a) surface, and b) cross-section of the GBSCC0911 pellet sintered at 1100 °C in air (i.e. the same conditions as the sample used in the electrical conductivity measurements).

Table S3. The comparison of structural data for different GBSCC compositions derived from DFT calculations and experiments.

material	calculated		experimental		references
	a [Å]	*c* [Å]	*a* [Å]	*c* [Å]	
$GdBaCo_2O_{5.5}$	3.76(1)	7.58(1)	3.8534	7.5318	[1]
	3.8363	7.6497			this work
$GdBaCo_2O_{5.5}$	3.838	7.676	3.88	7.542	[2]
$GdBaCoCuO_5$			3.875(2)	7.587(8)	[3]
	3.8830	7.5934			this work
$GdBa_{0.6}Sr_{0.4}Co_2O_{5.79}$			3.856	7.546	[4]
$GdBa_{0.4}Sr_{0.6}Co_2O_{5.83}$			3.840	7.549	[4]
$GdBaCoCuO_{5.643}$			3.894	7.604	[5]
$GdBa_{0.5}Sr_{0.5}CoCuO_{5.662}$			3.866	7.576	
$GdBa_{0.5}Sr_{0.5}CoCuO_{5.64}$			3.8625(1)	7.5809(1)	this work

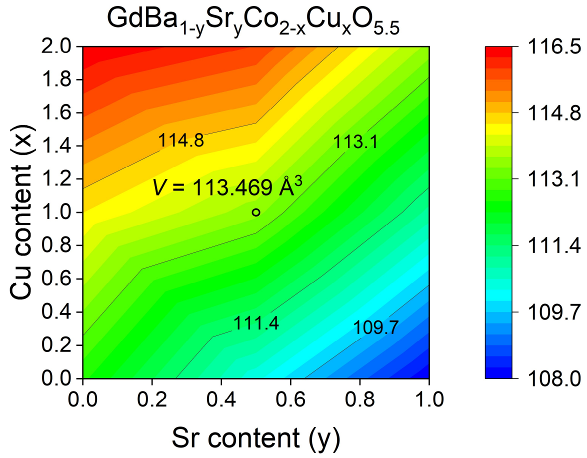

Figure S7. DFT-derived unit cell volume as a function of Sr and Cu content in $GdBa_{1-y}Sr_yCo_{2-x}Cu_xO_{5.5}$. Please notice that the same structural model was used for all calculations, despite that Cu-rich compositions could not be obtained as phase-pure.

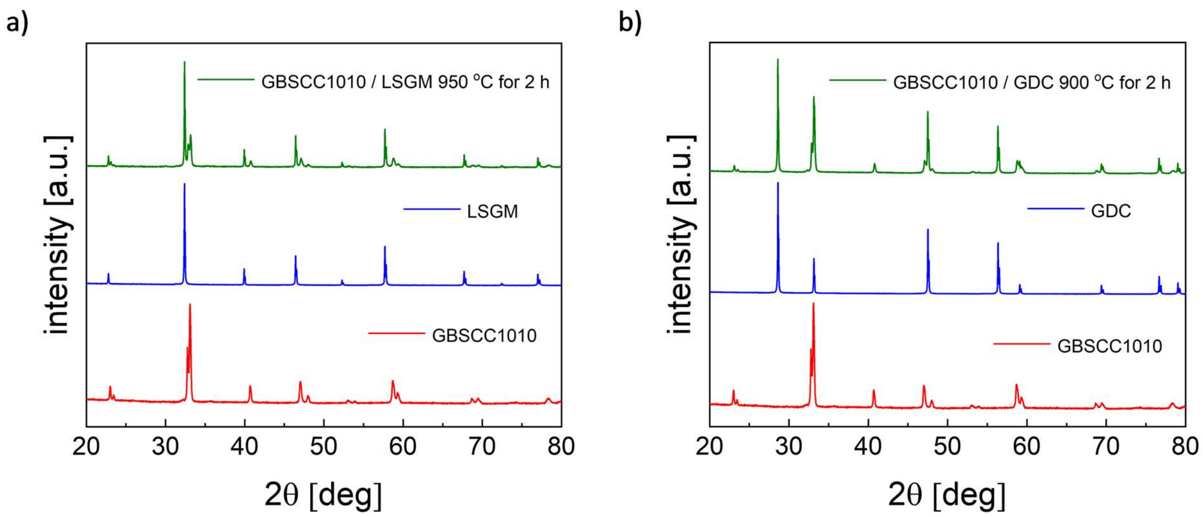

Figure S8. Results of XRD studies at RT for the annealed mixtures of GBSCC1010 and selected solid electrolytes. a) Data for GBSCC1010 and LSGM, b) data for GBSCC1010 and GDC.

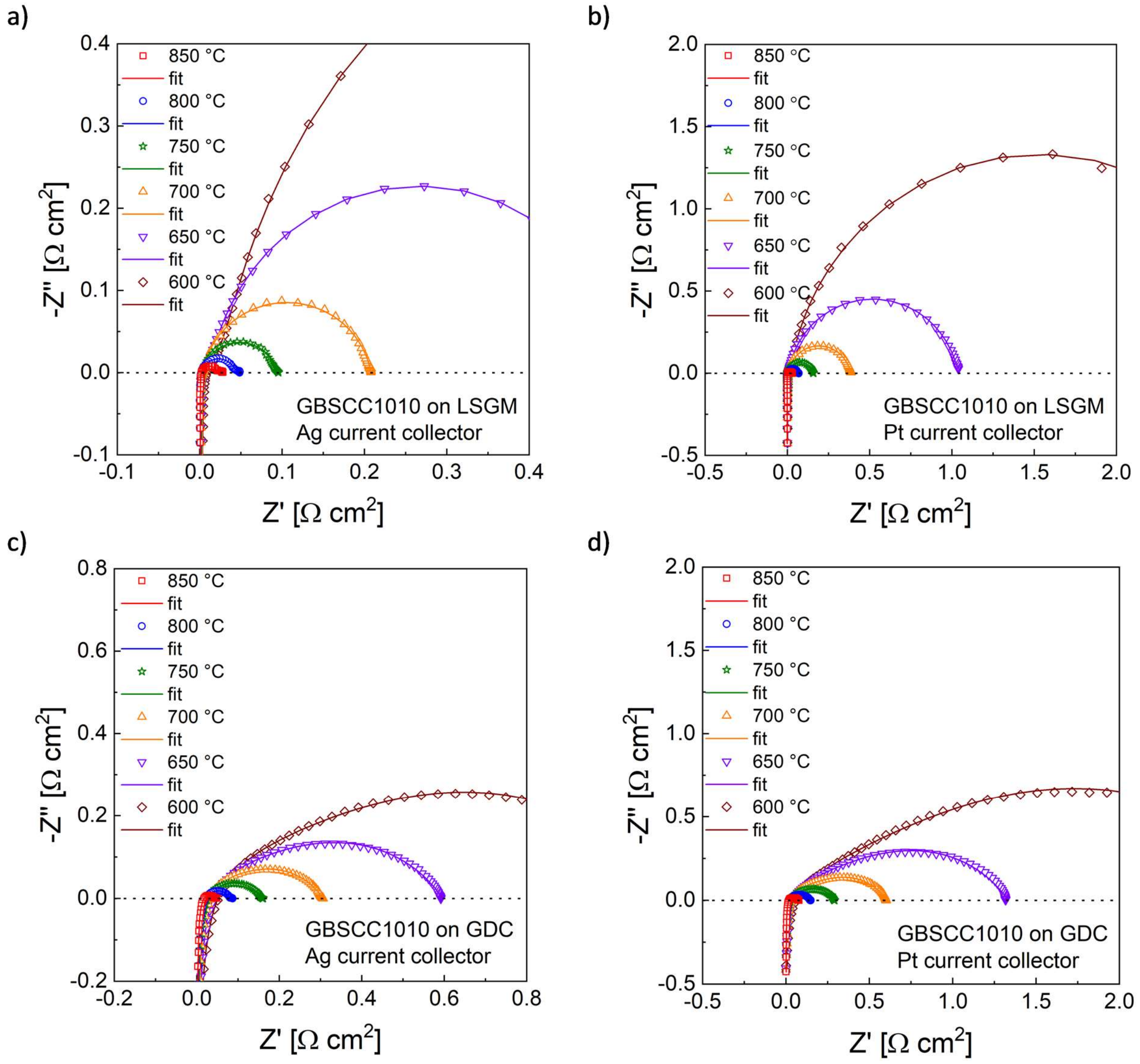

Figure S9. EIS data for the symmetrical cells with GBSCC1010 electrodes: a) LSGM-based with Ag, b) LSGM-based with Pt, c) GDC-based with Ag, d) GDC-based with Pt. Data recorded in the 600-850 °C temperature range. Respective fits are also shown.

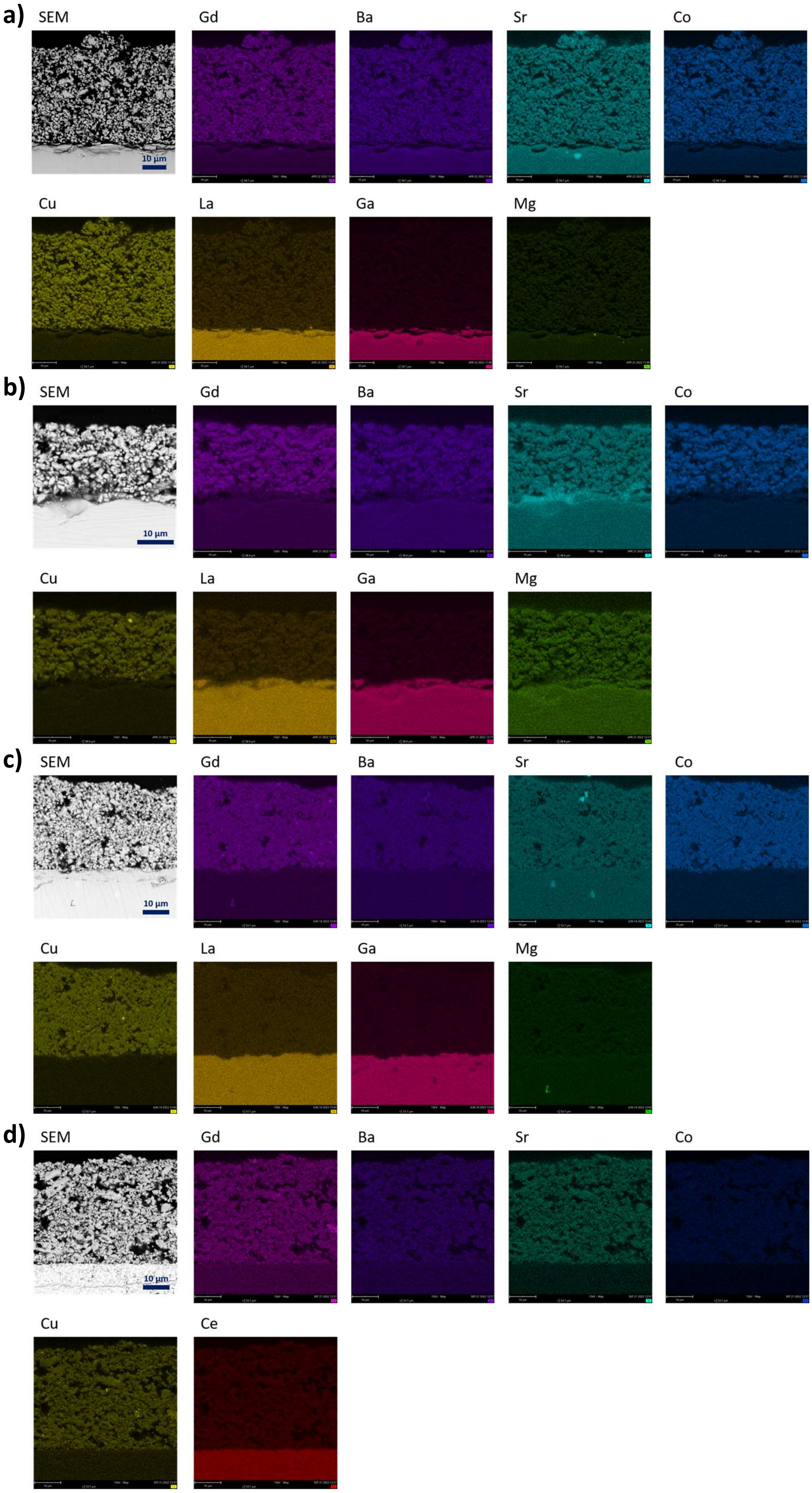

Figure S10. Morphology (cross-section) of the measured oxygen electrode together with the corresponding elemental maps obtained by EDX, a) GBSCC0911 after EIS tests in the

symmetrical cell (LSGM electrolyte), b) GBSCC1010 after long-term stability measurements in the symmetrical cell (LSGM electrolyte). Notice strontium segregation at the interface, c) GBSCC0911 after long-term stability measurements in the symmetrical cell (LSGM electrolyte). Sr segregation is suppressed, d) GBSCC0911 after long-term stability measurements in the symmetrical cell (GDC electrolyte). No Sr segregation occurred.

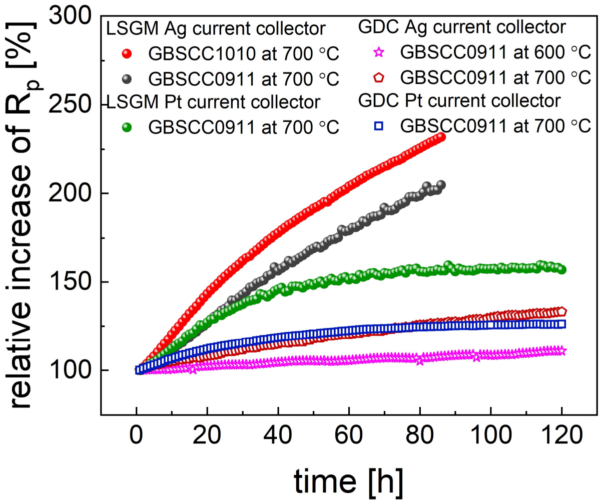

Figure S11. Relative increase with time of the polarization resistance of different cells (LSGM or GDC electrolyte) with GBSCC electrodes and Ag or Pt current collector.

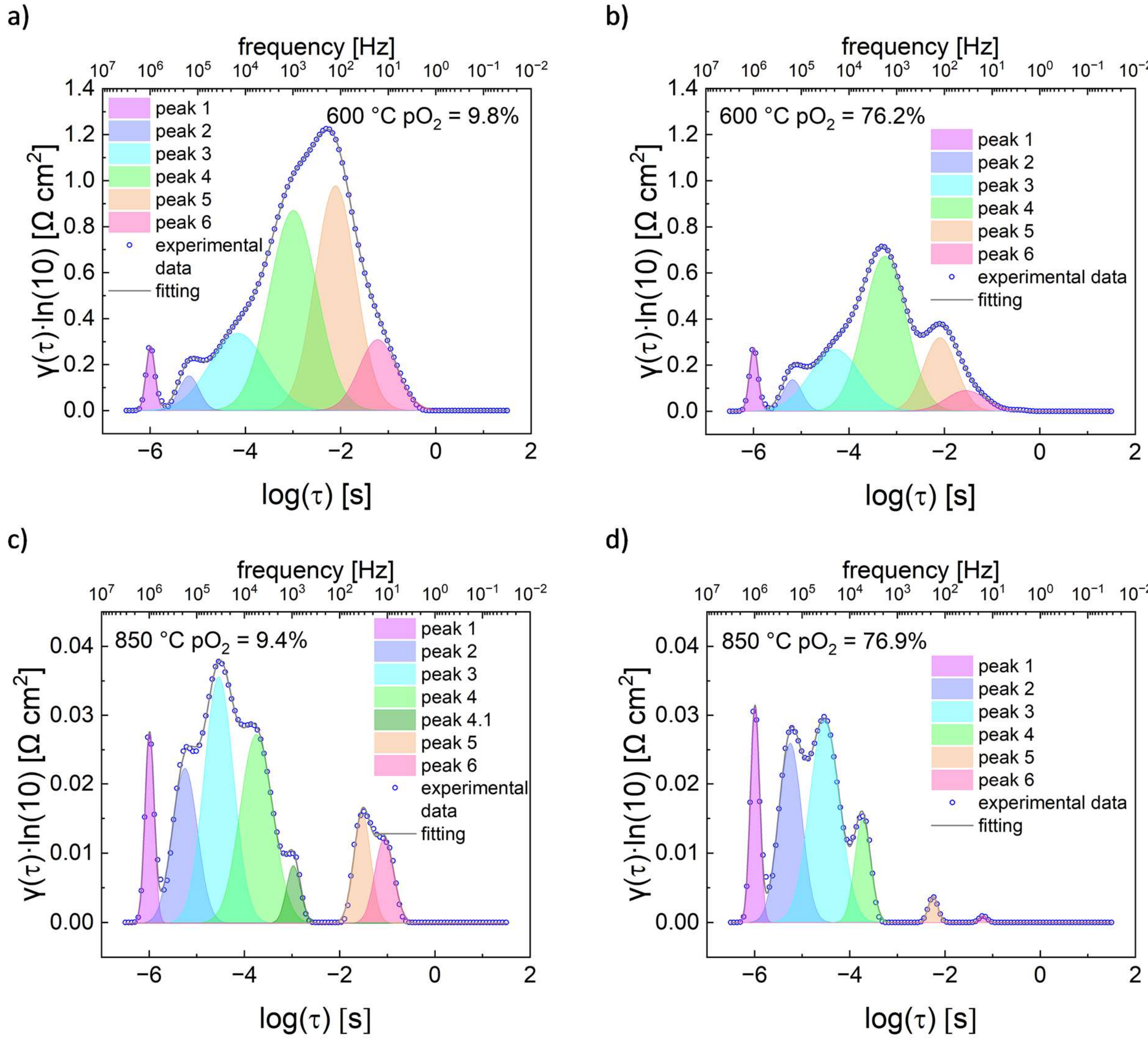

Figure S12. Results of DRT analysis for the selected pO_2 values as a function of τ, measured for the symmetrical cell with GBSCC0911 electrodes on GDC electrolyte and with Pt current collector: a-b) data at 600 °C and c-d) at 850 °C for different pO_2.

Table S4. Comparison of components of the total polarization resistance of symmetrical cells with GBSCC0911 electrodes and different electrolytes and current collectors. Data were recorded in air or close to air oxygen partial pressure at two different temperatures.

Cell	600 °C				850 °C			
	R_1 [Ω cm^2]	R_2 [Ω cm^2]	$R_{3,4}$ [Ω cm^2]	$R_{5,6}$ [Ω cm^2]	R_1 [Ω cm^2]	R_2 [Ω cm^2]	$R_{3,4}$ [Ω cm^2]	$R_{5,6}$ [Ω cm^2]
LSGM Ag	0.002	0.005	0.805	0.012	-	0.002	0.011	0.005
LSGM Pt	-	-	3.010	0.126	-	0.001	0.027	0.008
GDC Ag	0.064	0.091	0.849	0.049	0.006	0.010	0.017	0.007
GDC Pt	0.066	0.087	1.584	0.903	0.008	0.015	0.042	0.016

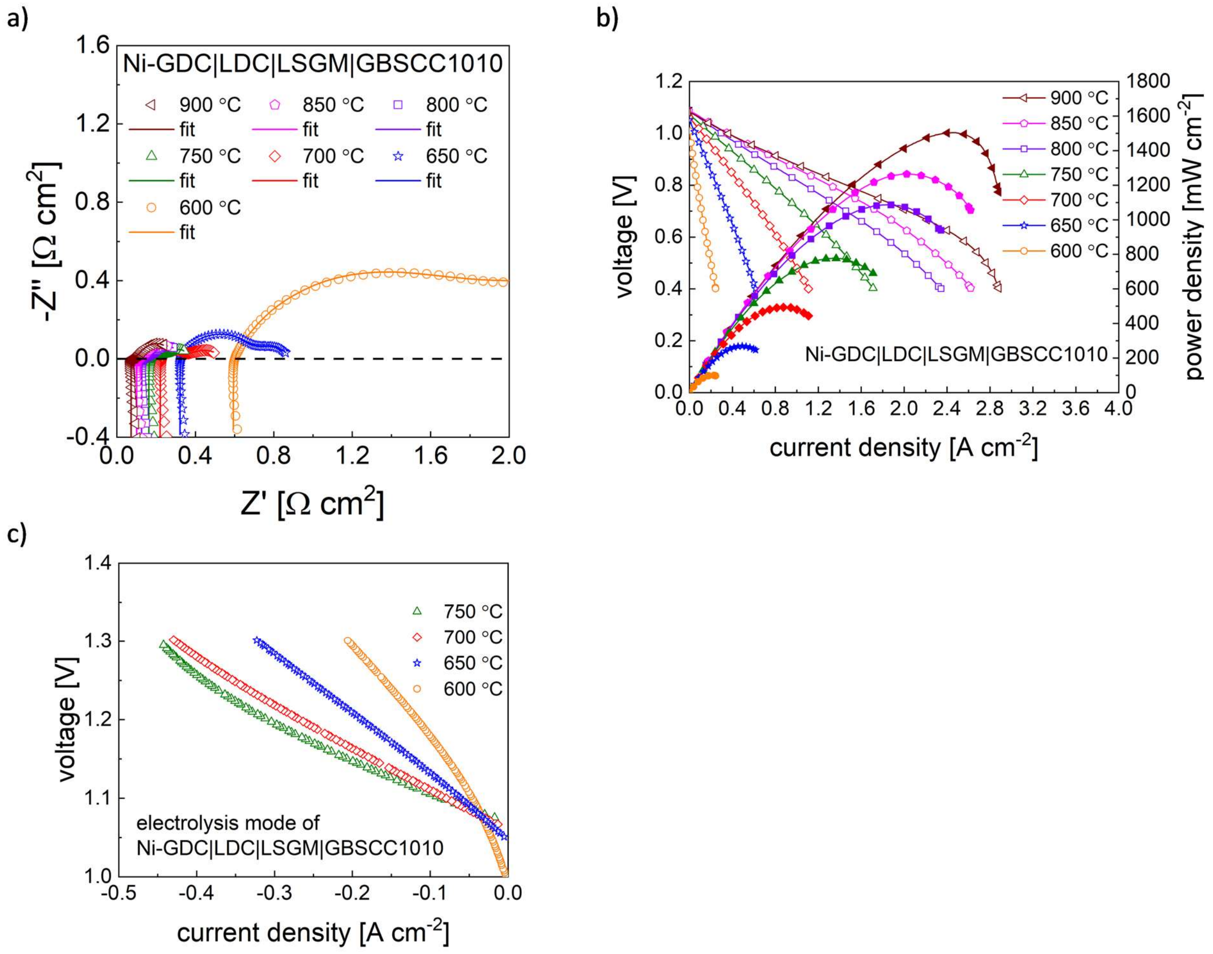

Figure S13. a) EIS data measured under open circuit conditions, b) voltage-current density (V-I) curves and power density output, c) performance in the electrolyzer mode for the Ni-GDC|LDC|LSGM|GBSCC1010 single cell. V-I curves measured for the humidified H_2 flow of 50 ml min^{-1}.

References

(1) Hermet, J.; Geneste, G.; Dezanneau, G. Molecular Dynamics Simulations of Oxygen Diffusion in $GdBaCo_2O_{5.5}$. *Appl Phys Lett* **2010**, *97* (17), 174102. https://doi.org/10.1063/1.3504250.

(2) Shiiba, H.; Nakayama, M.; Kasuga, T.; Grimes, R. W.; Kilner, J. A. Calculation of Arrangement of Oxygen Ions and Vacancies in Double Perovskite $GdBaCo_2O_{5+\delta}$ by First-Principles DFT with Monte Carlo Simulations. *Physical Chemistry Chemical Physics* **2013**, *15* (25), 10494. https://doi.org/10.1039/c3cp50316j.

(3) Shivakumara, C.; Hegde, M. S.; Subbanna, G. N. Synthesis, Structure and IR Absorption Studies of $LnBaCuCoO_5$ (Ln=Rare Earth) Oxides. *Bulletin of Materials Science* **1996**, *19* (4), 607-613. https://doi.org/10.1007/BF02745150.

(4) Kim, J.-H.; Prado, F.; Manthiram, A. Characterization of $GdBa_{1-x}Sr_xCo_2O_{5+\delta}$ ($0 \leq x \leq 1.0$) Double Perovskites as Cathodes for Solid Oxide Fuel Cells. *J Electrochem Soc* **2008**, *155* (10), B1023. https://doi.org/10.1149/1.2965792.

(5) West, M.; Manthiram, A. Layered $LnBa_{1-x}Sr_xCoCuO_{5+\delta}$ (Ln=Nd and Gd) Perovskite Cathodes for Intermediate Temperature Solid Oxide Fuel Cells. *Int J Hydrogen Energy* **2013**, *38* (8), 3364-3372. https://doi.org/10.1016/j.ijhydene.2012.12.133.
